# Stable colonization of *Akkermansia muciniphila* educates host intestinal microecology and immunity to battle against inflammatory intestinal diseases

**DOI:** 10.1038/s12276-022-00911-z

**Published:** 2023-01-04

**Authors:** Bin Wang, Xuheng Chen, Zhiyuan Chen, Huiwen Xiao, Jiali Dong, Yuan Li, Xiaozhou Zeng, Jinjian Liu, Guoyun Wan, Saijun Fan, Ming Cui

**Affiliations:** 1 Tianjin Key Laboratory of Radiation Medicine and Molecular Nuclear Medicine, Institute of Radiation Medicine, Chinese Academy of Medical Sciences and Peking Union Medical College, Tianjin, China; 2 The Key Laboratory of Biomedical Material, School of Life Science and Technology, Xinxiang Medical University, 453003 Xinxiang, China; 3 Department of Microbiology, College of Life Sciences, Nankai University, Tianjin, China

**Keywords:** Ulcerative colitis, Dysbiosis, Drug delivery, Nanoparticles

## Abstract

Gut microbial preparations are widely used in treating intestinal diseases but show mixed success. In this study, we found that the therapeutic efficacy of *A. muciniphila* for dextran sodium sulfate (DSS)-induced colitis as well as intestinal radiation toxicity was ~50%, and mice experiencing a positive prognosis harbored a high frequency of *A. muciniphila* in the gastrointestinal (GI) tract. Stable GI colonization of *A. muciniphila* elicited more profound shifts in the gut microbial community structure of hosts. Coexisting with *A. muciniphila* facilitated proliferation and reprogrammed the gene expression profile of *Lactobacillus murinus*, a classic probiotic that overtly responded to *A. muciniphila* addition in a time-dependent manner. Then, a magnetic-drove, mannose-loaded nanophase material was designed and linked to the surface of *A. muciniphila*. The modified *A. muciniphila* exhibited enhancements in inflammation targeting and intestinal colonization under an external magnetic field, elevating the positive-response rate and therapeutic efficacy against intestinal diseases. However, the unlinked cocktail containing *A. muciniphila* and the delivery system only induced negligible improvement of therapeutic efficacy. Importantly, heat-inactivated *A. muciniphila* lost therapeutic effects on DSS-induced colitis and was even retained in the GI tract for a long time. Further investigations revealed that the modified *A. muciniphila* was able to drive M2 macrophage polarization by upregulating the protein level of IL-4 at inflammatory loci. Together, our findings demonstrate that stable colonization of live *A. muciniphila* at lesion sites is essential for its anti-inflammatory function.

## Inflammatory bowel disease: Magnets help modified bacteria colonize the gut

A modified gut bacterium that can colonize the gut more effectively with the help of magnets could offer a novel treatment for inflammatory bowel diseases (IBD). In experiments on mouse models, Ming Cui at the Chinese Academy of Medical Sciences and Peking Union Medical College, China, and co-workers showed that when the bacterium *Akkermansia muciniphila* successfully colonizes the gut it repairs damage caused by IBD and boosts the proliferation of another probiotic bacterium, *Lactobacillus murinus*. To enhance colonization chances, the team added Fe_3_O_4_ nanoparticles to the bacterium as well as mannose, a sugar monomer that is drawn to the macrophages present in inflamed regions. The magnetic *A. muciniphila* was then attracted to the correct part of the gut and held in place using magnets on the skin. Initial experiments suggest that this safely enhances stable colonization.

## Introduction

Inflammatory bowel diseases (IBDs), such as Crohn’s disease and ulcerative colitis, are progressive immune-mediated diseases in the large intestine, affecting ~6.8 million patients worldwide with increasing incidence and need for remedy^1,2^. Owing to the sensitivity of the small intestine to irradiation, abdominal and pelvic cancer patients always suffer from intractable small intestine toxicity following radiotherapy^3^. Both the small and large intestines harbor trillions of microorganisms collectively termed the gut microbiota, which help educate and tune the balance of energy metabolism and the immune system of hosts^4,5^. Dysbiosis in gut microbiota precipitates the occurrence of multiple intraintestinal diseases^2,6^. The developments of IBD and radiation intestinal toxicity intertwine with changes in the gut microbiota configurations and metabolite profiles^7^. In light of these findings, therapeutic strategies focused on fighting diseases through reversing the microecology in the GI tract, such as fecal microbiota transplantation (FMT) and microbial preparations, have experienced a renaissance in preclinical or clinical scenarios^8–10^. Although some clinical trials report advantages in disease intervention and treatment, the benefit of microbiota-based therapy remains short-term with mixed success in clinical applications^11–13^.

FMT and antibiotics are the most effective options to regulate the gut microecology^8,14^. FMT has been proven by a large number of clinical trials to be efficacious and safe for ulcerative colitis alleviation^15,16^, but its remission rate for ulcerative colitis is still low in adults (<30%)^17^. Increasing the dose or frequency of FMT only limitedly improves the remission rate for IBD but is entwined with elevation of adverse events^18^. Probiotic combinations or monobacterial preparations also show beneficial effects in reducing insulin resistance in patients with type 2 diabetes clinically^19^. However, the efficacy of probiotic therapy, especially for monobacterial preparations, is often restricted owing to the genetic characteristics and gut microbial signature of hosts^20–22^. In line with FMT, higher doses or more frequent intake of probiotics also increase the risk of dysbiosis in the gut microbiome as well as add time and financial burden^11^. Anaerobic probiotics are likely to accumulate in inflammation sites with hypoxia in the GI tract^23^, but the tendency is very weak due to intestinal motility, impairing the retention of probiotics at the lesion. *Akkermansia muciniphila* (*A. muciniphila*) is a potential probiotic and has been proven to fight against colorectal cancer and many metabolic diseases^24,25^. In our previous study, although *A. muciniphila* alleviated intestinal radiation injuries in mouse models, some individuals always experienced unsatisfactory therapeutic effects^26^. Whether the colonization of probiotics dictates their therapeutic efficacy and how monobacterial preparation optimizes the gut microhabitats remain poorly understood.

In the present study, we set out to explore the relationship between *A. muciniphila* colonization in the GI tract and its therapeutic efficacy against inflammatory GI diseases in mouse models. Then, the intestinal colonization of *A. muciniphila* is improved by the assistance of a magnetic delivery system, which promotes the response rate and therapeutic efficacy of *A. muciniphila* in the treatment of inflammatory GI diseases. The benefits of enhanced colonization are investigated in terms of modulating the gut microecology and exciting host immune responses. Together, our findings provide novel insight into gut microbial preparation-based therapy and technical support to overcome the limitations of probiotic therapy in preclinical settings.

## Materials and methods

### Mice

Seven-week-old male C57BL/6J mice (~19 g) were purchased from Beijing SiBeiFu Bioscience Co. Inc. (Beijing, China). Mice were housed in the specific pathogen-free level animal facility at the Institute of Radiation Medicine (IRM), the Chinese Academy of Medical Sciences (CAMS), and maintained in an enriched environment with a temperature-controlled room in a 12 h light–dark cycle, with food and water available. Before the experiment, the mice were allowed to adapt to the experimental environment for 1 week. Then, all the mice were orally administered 3% dextran sodium sulfate (DSS) in drinking water ad libitum for 8 days in total. The mice with obvious symptoms were selected for further study. Briefly, the mice were grouped evenly according to the disease activity index (DAI) score (see Supplementary Table 1) at Day 5, and each group then received different treatments from Days 6 to 8. Before sacrifice at Day 10, the mice were exposed to clean water for 1 day. Animal experiments were performed according to the institutional guidelines approved by the Animal Care and Ethics Committee of IRM-PUMC (the ethical approval number is IRM-DWLL-2020039).

### Radiation study

A Gammacell-40 ^137^Cs irradiator (Atomic Energy of Canada Limited, Chalk River, ON, Canada) at a dose rate of 0.88 Gy per minute was used for radiation experiments. All experimental mice received 12 Gy γ-ray total abdominal irradiation (TAI). Then, the mice in the treatment group were orally gavaged with *A. muciniphila* or AKK@MFe_3_O_4_. On the 21st day following radiation exposure, the mice were sacrificed, and then the small intestine (distal tissue and mid tissue for protein and RNA extraction, respectively, and proximal tissue for histology) and serum were collected for further analyses.

### *A. muciniphila*

*A. muciniphila* MucT (ATCC BAA-835) was cultured in liquid brain heart infusion (BHI) medium supplemented with mucin in a strict anaerobic incubator (90% N_2_ and 10% H_2_) at 37 °C. *A. muciniphila* was precultured, followed by a fermentation step. The relationship between the plate count results and OD_600_ values was established first. Briefly, 100 μl from 1 ml of bacterial suspension (dispersed in anaerobic PBS) was taken for OD_600_ value measurement, and the OD_600_ = 0.8 was equivalent to 1 × 10^8^ CFU/ml bacteria. For the fermentation step, 1 × 10^5^ CFU bacteria were inoculated into 20 ml of liquid medium and fermented for 5 h (the exponential phase), followed by centrifugation at 3000 *rpm* for 5 min and resuspension with anaerobic PBS to OD_600_ = 0.8 to determine the amount of bacteria. To explore the effects of *A. muciniphila* on colitis, the mice with DSS-induced acute colitis were administered *A. muciniphila* (5 × 10^7^ CFU/200 μl per mouse) orally for 3 days, while the corresponding control was given sterile PBS with an equivalent volume. For treatment with *A. muciniphila* at a high concentration, the bacteria were diluted with anaerobic PBS to a final concentration of 2.5 × 10^8^ CFU/200 μl per mouse.

### Grouping standard for “poor response” and “response” mice

“Poor response (PR)” and “response (R)” mice were distinguished following *A. muciniphila* treatment. The grouping standards for PR (mice with higher scores) and R (mice with lower scores) mice were based on previous reports with slight changes^27^. The criteria for the scores are provided in Supplementary Table 1.

### In vitro cocultivation of *L. murinus* and *A. muciniphila*

*Lactobacillus murinus* (CGMCC 1.2306, *L. murinus*) was cultured in lactobacilli MRS agar under a strict anaerobic incubator (90% N_2_ and 10% H_2_). *L. murinus* was also precultured, followed by a fermentation step. *L. murinus* at the exponential phase (fermenting for 24 h) was used for the experiment. Similarly, the relationship between the plate count results and OD_600_ values of *L. murinus* was established first. One hundred microliters from 1 ml of bacterial suspension (dispersed in anaerobic PBS) was taken for OD_600_ value measurement, and the OD_600_ = 0.7 was equivalent to 1 × 10^8^ CFU/ml bacteria. *A. muciniphila* (0 or 2 × 10^8^ CFU) at the exponential phase was inoculated into solid BHI medium supplemented with mucin. Then, *L. murinus* (5 × 10^7^ CFU) dispersed in 100 μl anaerobic PBS was added to the petri dish (10 × 10 cm) culturing *A. muciniphila*, and the two kinds of bacteria were separated by a layer of cellophane (only small molecule compounds were allowed to transit). There were three cocultivation strategies: *A. muciniphila* (0 CFU) for 6 h; *A. muciniphila* (0 CFU) for 5 h followed by *A. muciniphila* (2 × 10^8^ CFU) for 1 h; and *A. muciniphila* (2 × 10^8^ CFU) for 6 h. Following cocultivation, the cellophane was removed, and *L. murinus* cultured on the cellophane was collected using a sterile cotton brush. The sample was stored at −80 °C for mononuclear transcriptome sequencing.

### Preparation and characterization of MFe_3_O_4_

The Fe_3_O_4_ nanoclusters were prepared by the hydrothermal method according to a previous report with slight changes^28^. More details are presented in the supplementary materials.

### Preparation of AKK@MFe_3_O_4_

AKK@MFe_3_O_4_ was prepared through chelation between the sulfhydryl group and MFe_3_O_4_ (Supplementary Fig. 7a). Simply, live *A. muciniphila* was washed three times with PBS to remove metabolites in the culture medium as much as possible, and then the surface of *A. muciniphila* was first functionalized with sulfhydryl groups by Traut’s reagent (the reaction efficacy was ~60–70%)^29–31^. The sulfhydrylated *A. muciniphila* (1 × 10^8^ CFU/ml) was coincubated with MFe_3_O_4_ of different Fe_3_O_4_ concentrations (0, 50, 100, 150, 200 μg/ml) under gentle shaking in an oxygen-free environment (room temperature) for 2 h. The obtained AKK@MFe_3_O_4_ was separated (3000 rpm, 5 min), washed three times using PBS, and finally stored at −80 °C in PBS containing 25% glycerol for further use. Importantly, both the magnetic adsorption characteristics and viability of AKK@MFe_3_O_4_ were detected before freezing and after thawing.

### Preparation of heat-killed AKK@MFe_3_O_4_

The live *A. muciniphila* was washed three times with PBS to remove metabolites in the culture medium as much as possible, then heated at 100 °C for 15 min and washed with anaerobic PBS three times again^32^. The heat-killed *A. muciniphila* was then modified with MFe_3_O_4_. The obtained heat-killed AKK@MFe_3_O_4_ was separated and washed three times using anaerobic PBS.

### Magnetic drive test

To verify the retention efficiency of AKK@MFe_3_O_4_, the magnetic stability and viability were first investigated in simulated gastrointestinal (GI) environments in vitro. AKK@MFe_3_O_4_ (1 × 10^8^ CFU of *A. muciniphila*) was dispersed in 1 ml of simulated gastric juice (pH 3.0) and intestinal fluid (pH 7.8). Then, the mixture was attracted by a magnet (the suction power was 2 kg, and the surface magnetism was 1000 Gauss) with a 1 cm interval. The device was placed in a 37 °C shaker at 65 rpm, and the adsorption of AKK@MFe_3_O_4_ was observed at predetermined time points. For the viability test, 10 μl AKK@MFe_3_O_4_ (1 × 10^6^ CFU of *A. muciniphila*) treated with different pH values was inoculated into 1 ml liquid BHI medium and cultured for 12 h. Then, the precipitate agglomerate of *A. muciniphila* was observed, and the OD_600_ value (resuspended with PBS) was measured.

Next, the in vivo retention effect of AKK@MFe_3_O_4_ was evaluated *via* an in vivo imaging system. Briefly, AKK@MFe_3_O_4_ (1 × 10^8^ CFU of *A. muciniphila*) or *A. muciniphila* (1 × 10^8^ CFU) was preincubated with the DIR fluorescence probe (30 μM) as previously described^33,34^ and washed with PBS three times. Then, the mice were orally treated with 200 μl DIR-labeled AKK@MFe_3_O_4_ (5 × 10^7^ CFU of *A. muciniphila* per mouse) and DIR-labeled *A. muciniphila* (5 × 10^7^ CFU per mouse). After the oral treatment, the mice were fasted and waterless and accepted magnet attraction for 3 h when the bacteria arrived at the specific position of the mouse intestine (small intestine or colon). When the magnet attraction was completed or several hours after the completion, the mice were sacrificed, and the intestines were harvested for imaging by the in vivo imaging system.

### In vivo magnet attraction

To preserve the normal movement state of mice as much as possible, a wearable bag made of soft plastic was designed. The hairs on the back and abdomen of the experimental mice were shaved. Then, the bag with a magnet was adhered to the skin of the mouse for every treatment (Supplementary Fig. 8a). The device was stable during the experimental period (3 h) and removed after each treatment. The mice were awake, and their movements were not significantly affected (see supplementary video), which guaranteed normal GI motility. All groups of mice were equipped with the magnetic device at the time of treatment to minimize the effects of the device on the experimental results.

### Tissue collection

DAI score analysis was used to assess the severity of colitis as described in Supplementary Table 1. After the mice were sacrificed, the colon length and spleen weight were measured. Then, the colons were collected for RNA isolation (distal tissue), protein extraction (proximal tissue) and histological staining (mid tissue).

### Relative quantification of *A. muciniphila* by q-PCR

For the relative abundance analysis of *A. muciniphila* in the colon or small intestine, the mouse colons or small intestines were cut lengthwise, and the contents were gently removed. Then, the mucus layer was scraped off with a sterile cotton brush for the extraction of bacterial DNA using a TIANamp oral DNA kit (TIANGEN, Beijing, China). Finally, q-PCR was used to assess the number of *A. muciniphila* as previously reported^23^. The primers are listed in Supplementary Table 2.

### Quantification of the expression of IL-1β, IL-4, IL-6, IL-10 and TNF-α by ELISA

Colon or small intestine tissues in each experimental group were ground up with 400 μl saline per 0.1 g, followed by centrifugation for 10 min at 4000 *rpm* and 4 °C. Proteins in the supernatant were measured by corresponding ELISA kits (Mlbio, Shanghai, China) according to the manufacturer’s instructions. Optical density was read at 450 nm (Rayto, Shenzhen, China).

### RNA isolation and quantitative reverse transcription real-time PCR (qRT‒PCR)

Total RNA was extracted from intestine tissues using an Animal Tissue Total RNA Extraction Kit (TIANGEN, Beijing, China). Complementary DNA was synthesized from total RNA using poly (A)-tailed total RNA and reverse transcription primers with ImPro-II Reverse Transcriptase (Promega, Madison, WI, USA) according to the manufacturer’s protocol. qRT‒PCR was performed according to the instructions of Fast Start Universal SYBR Green Master Mix (Rox) (Roche Diagnostics GmbH, Mannheim, Germany). Relative transcriptional folds were calculated as 2^−ΔΔCt^. GAPDH was used as an internal control for normalization. The primers are listed in Supplementary Table 2.

### Fluorescein isothiocyanate (FITC)-dextran permeability experiments

The radiated mice with the indicated treatments were fasted overnight (12 h) and then administered FITC-dextran (Sigma‒Aldrich, Spain) (60 mg per 100 g mouse body weight) in a volume of 200 μl at 21 d following TAI. Four hours after FITC-dextran administration, the blood of mice was collected from the eye socket and centrifuged at 1500 *rpm* for 15 min to obtain the serum. The fluorescence intensity of each serum sample (100 μl) was measured by enzyme-linked immunoassay (DTX 880 Multimode Detector, USA).

### Immunostaining of mucins and localization of *A. muciniphila* by fluorescent in situ hybridization (FISH)

Following euthanasia, the colon tissues (containing fecal material) were cut into pieces and fixed in methanol-Carnoy’s fixative (methanol: chloroform: glacial acetic acid = 6:3:1) overnight at room temperature and then embedded in paraffin. Tissues were sectioned at 5 μm thickness, and FISH was performed according to standard protocols^35^. The *Akkermansia*-specific probe sequence was CCTTGCGGTTGGC-TTCAGAT, which was 5′-labeled with Cy5. Finally, slides were mounted using prolonged antifade mounting media containing DAPI (Biosharp), and the observations were performed with a Leica microsystem.

### Bacterial diversity analysis

Stool samples were freshly collected from three independent experiments and stored at −80 °C until use. Briefly, we collected fecal pellets from all the numbered experimental mice at the end of each replicate experiment. Three stool samples were randomly selected from each group (PBS, R and PR) in each replicate of the experiment, which constituted the overall sample used for final gut microbiota analysis. The primers are listed in Supplementary Table 2. More details are presented in the supplementary materials.

### Transcriptome sequencing

*L. murinus* cocultured with *A. muciniphila* was collected, and RNA was extracted using standard extraction methods. RNA degradation and contamination were monitored on 1% agarose gels, and RNA integrity was assessed using the RNA Nano 6000 Assay Kit of the Bioanalyzer 2100 system (Agilent Technologies, CA, USA). More details are presented in the supplementary materials.

### Statistical analysis

The Kolmogorov‒Smirnov test and Levene test were used to verify that all data were normally distributed and had homogeneity of variance. Significance was determined using Student’s *t test*s or one-way ANOVA corrected for multiple comparisons with an LSD-t test (SPSS software, version 20.0). The statistical tests in the study of microbial diversity were assessed by the Wilcoxon rank sum test and Tukey HSD test. Each experiment was repeated at least three times. The data are presented as the means ± SDs with respect to the number of samples (n) in each group. The significance level was set at 0.05 for all tests. No prior power analysis was used to predetermine the sample size.

More details are presented in the supplementary materials.

## Results

### The colonization of *A. muciniphila* in the GI tract dictates its therapeutic efficacy in DSS-induced colitis

**Fig. 1:**
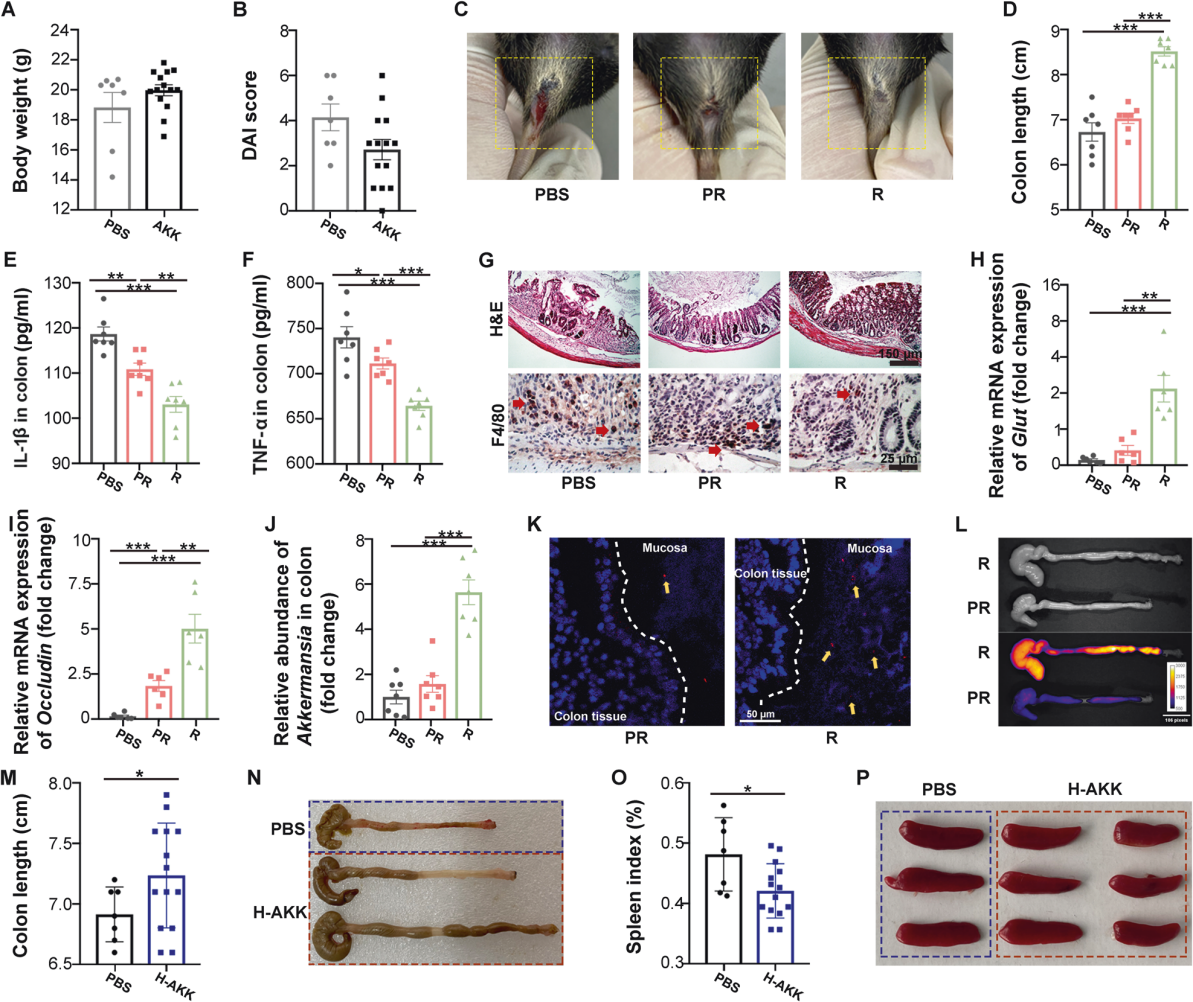
A. *muciniphila* colonization correlates with its therapeutic efficacy in DSS-induced colitis. Groups: PBS, the mice were administered PBS, *n* = 7; AKK, the mice were administered *A. muciniphila* (5 × 10^7^ CFU), *n* = 14; PR, poor response to *A. muciniphila* treatment, *n* = 7; R, response to *A. muciniphila* treatment, *n* = 7; H-AKK, the mice were administered *A. muciniphila* at a high dose (2.5 × 10^8^ CFU). **A** The body weight of mice at Day 10. **B** The DAI score of colitis assessed at Day 10. **C** Representative photographs of rectal bleeding of IBD mice at Day 10. **D** Statistical results of colon length of mice with the indicated treatments. **E**, **F** The levels of IL-1β and TNF-α in inflamed colon homogenate of mice were measured by ELISA. **G** The morphologies of the colons from mice in the three groups were shown by (**H** and **E**)(the first line, scar bar: 150 μm), and the infiltrations of macrophages in inflamed colons were shown by IHC of F4/80 (the second line, the red arrows pointed to the macrophages, scar bar: 25 μm). **H**, **I** The relative mRNA expression levels of *Glut* and *Occludin* were examined in colon tissues from mice by qRT‒PCR. **J** The relative abundance of *A. muciniphila* in the colonic mucosa was detected by q-PCR. **K** Confocal microscopy analysis of *A. muciniphila* localization: bacteria, red; DNA, blue. The yellow arrow points to the bacteria (scar bar: 50 μm). **L** Distributions of *A. muciniphila* in the colons of mice with colitis. The mice were orally administered DIR-labeled *A. muciniphila* for 3 days, and fluorescence images of the colons were obtained at 6 h following the last treatment. **M** Statistical results of colon length of mice. **N** Representative photographs of the colons of mice. **O** Statistical results of the spleen index of mice. **P** Representative photographs of the spleens of mice. **A**, **B**, **M** and **O** Significance was determined using Student’s *t test*; (**D**–**J**) Significance was determined using one-way ANOVA corrected for multiple comparisons with an LSD-t test; *P < 0.05, **P < 0.01, ***P < 0.001. Data are the mean ± SD.

In line with previous clinical trials, visible individual differences in therapeutic efficacy were observed when we applied *A. muciniphila* to mitigate DSS-induced colitis in mice. As shown in Fig. 1A, B and Supplementary Fig. 1a, approximately two-thirds of mice poorly responded to *A. muciniphila* replenishment (5 × 10^7^ CFU/mouse), as judged by no significant improvement in body weight and DAI score. Then, the *A. muciniphila*-treated mice were divided into “Response” (R) and “Poor Response” (PR) groups according to body weight, DAI score and colon length (Fig. 1C, D and Supplementary Fig. 1b, c). Although all the mice experienced catabatic colonic injuries, the mice in the R group had a lower inflammation status, shorter wound bed length and better intestinal integrity than their counterparts in the PR group (Fig. 1E, F, first line in g, h and i). In addition, immunohistochemical (IHC) staining of colonic F4/80 showed that the mice in the R group had less infiltration of macrophages in the inflammatory lesion (Fig. 1G, second line). To determine the relationship between *A. muciniphila* colonization and therapeutic efficacy, qPCR assays were performed and showed that the mice in the R group harbored approximately six times more *A. muciniphila* than those in the PR group (Fig. 1J). In addition, fluorescence in situ hybridization (FISH) assays further validated more *A. muciniphila* in the colonic mucosa from mice in the R group (Fig. 1K). To visualize the dynamics of *A. muciniphila* after oral treatment, *A. muciniphila* was labeled by DIR and then administered to mice orally, and the intestines were imaged by an in vivo imaging system. In general, most *A. muciniphila* remained in the large intestine of mice with colitis for ~1 h and was excreted within 5 h after oral administration (Supplementary Fig. 2a). Although the intestinal dynamics of *A. muciniphila* showed no significant difference between the PR and R groups at early time points (0.5 h and 1 h) after a single oral gavage, the DIR-labeled *A. muciniphila* seemed to be retained more within 5 h in the R group (Supplementary Fig. 2b). In addition, compared to mice in the PR group, mice in the R group harbored more labeled *A. muciniphila* in the large intestine after 3 consecutive days of administration (Fig. 1L). All the above evidence indicated that a greater *A. muciniphila* abundance correlated with better colitis remission. Next, we increased the concentration of *A. muciniphila* by oral gavage (from 5 × 10^7^ to 2.5 × 10^8^ CFU/200 μl/mouse), but no more positive responders appeared (Fig. 1M–P and Supplementary Fig. 2c), indicating that the increased amount of *A. muciniphila* hardly improves the therapeutic efficacy to DSS-induced colitis.

### *A. muciniphila* shapes the gut microbiota configurations in a colonization-dependent manner

**Fig. 2:**
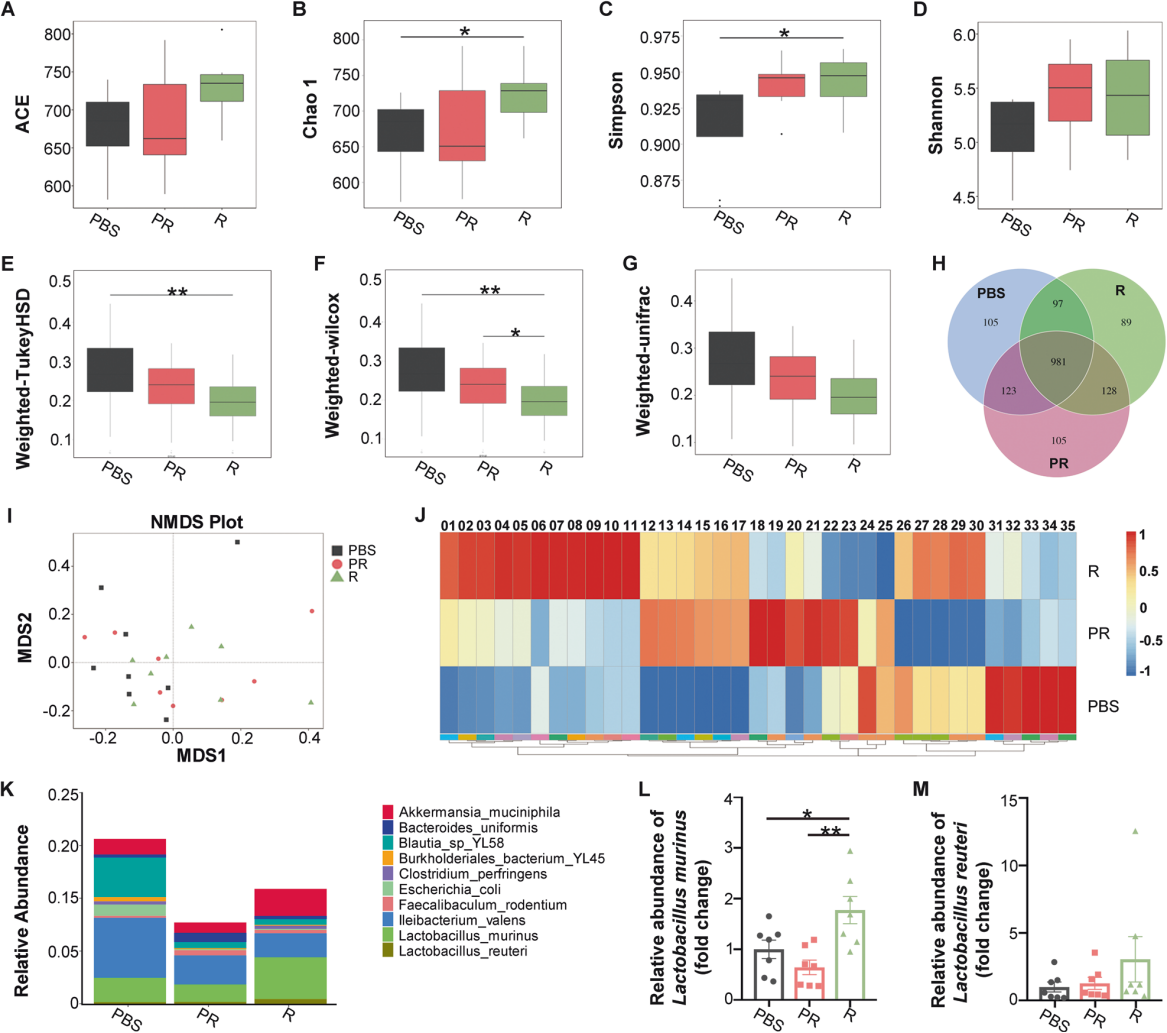
The colonization intensity of *A. muciniphila* affects the gut microecology of mice. **A–D** Alpha diversity of fecal microflora was measured: (**A**) ACE diversity index, (**B**) Chao1 diversity index, (**C**) Simpson diversity index and (**D**) Shannon diversity index. The numbers in the histograms represent the abundance of microbial species in each individual. **E–G** Beta diversity of fecal microflora: (**E**) Weighted-Tukey HSD diversity index, (**F**) Weighted-Wilcox diversity index and (**G**) Weighted UniFrac index. **H** Venn diagram of the number of bacterial species, and the numbers represent the abundance of microbial species of all individuals in each group. **I** NMDS analysis was performed to assess the alteration of gut bacterial taxonomic profiles in the three groups. **J** The difference heatmap of gut bacterial composition at the phylum level. Notes for the numbers are shown in Supplementary Table 3. **K** The relative abundance of the top 10 most varied bacterial strains at the species level (no others, outliers removed). **L**, **M** Statistical results of the relative abundance of *Lactobacillus murinus* (**L**) and *Lactobacillus reuteri* (**M**) in the mouse feces in the three groups. **L**, **M** Significance was determined using Student’s *t test*; *P < 0.05, **P < 0.01. Data are the mean ± SD.

Then, the gut microbiota configurations of mice in different colonization contexts were investigated by fecal 16 S rRNA gene sequencing. As expected, mice in the R group showed more overt alterations in both the α- and β-diversity of the fecal microbiota than mice in the PR group (Fig. 2A–G). The mice in the R group harbored the lowest total number of bacterial species in their feces and shared fewer bacterial species (981 + 97) with control mice compared to the PR group (981 + 123) (Fig. 2H). Although the NMDS plot showed a slight shift in fecal bacteria of mice among the three cohorts, the heatmap indicated the detailed alterations in taxonomic proportions and predicted functions of gut bacteria at the phylum level (Fig. 2I, J, and Supplementary Fig. 3a). Specifically, *A. muciniphila* retention was associated with an increased relative abundance of probiotics such as *Lactobacillus reuteri*, especially for *Lactobacillus murinus* (*L. murinus*) species (Fig. 2K–M and Supplementary Fig. 3b, c). Together, our results suggest that *A. muciniphila* might educate the gut microbiota community in a colonization-dependent manner and that stable colonization of *A. muciniphila* may be required to regulate gut microecology.

### *A. muciniphila* reprograms the gene expression profiles of neighboring bacteria in a time-dependent manner

**Fig. 3:**
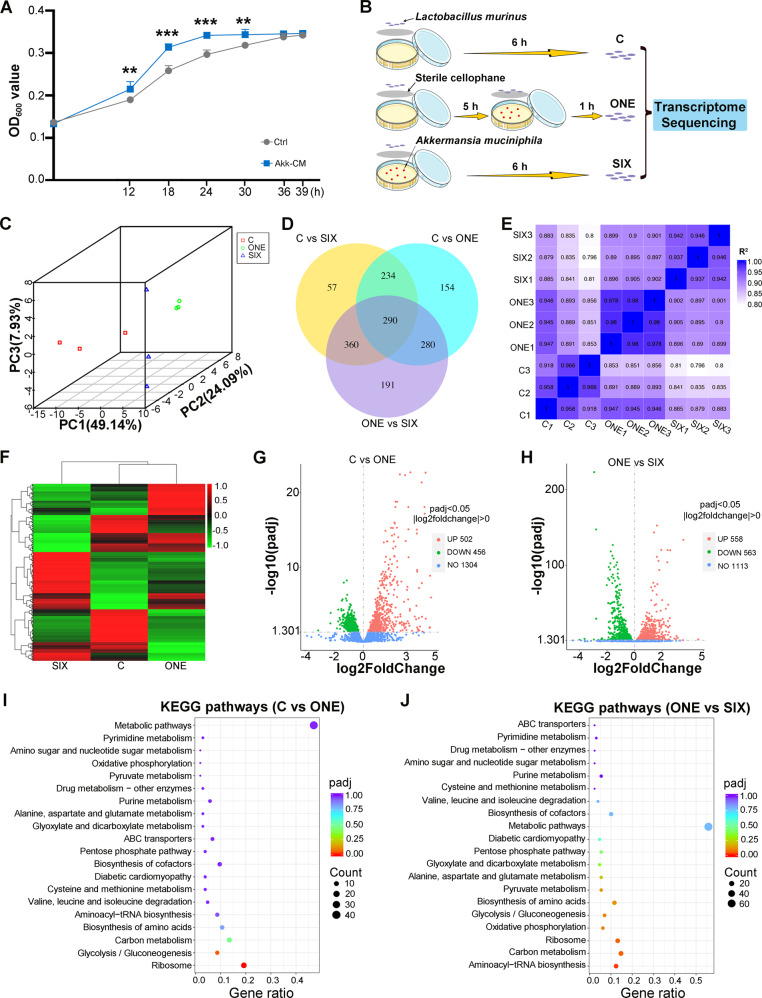
*A. muciniphila* reprograms the gene expression profile of *L. murinus* in vitro. **A** The growth rate of *L. murinus* cultured in clean MRS medium (Ctrl) and MRS medium containing metabolites of *A. muciniphila* (AKK-CM). Significance was determined using Student’s *t test*; **P < 0.01, ***P < 0.001. Data are the mean ± SD. **B** Scheme of cocultivation of *L. murinus* and *A. muciniphila*. **C** PCA was performed to assess the alteration of the gene profile from *L. murinus* in the three groups. **D** Venn diagram composed of the differentially expressed genes between different comparison combinations. **E** Pearson correlation between samples; each group included three independent samples. **F** Heatmap of differential gene cluster analysis in the three groups. The colors in the heatmap can only be compared horizontally (the expression of the same gene in different samples) but cannot be compared vertically (the expression of different genes in the same sample). In horizontal comparison, red indicates high gene expression, and blue indicates low gene expression. **G, H** Volcano map of differentially expressed genes (**C** vs. ONE, **H**; ONE vs. SIX, **I**). **I**, **J** KEGG pathway enrichment analysis of the differentially expressed genes with upregulation in the ONE group compared with the C group (**I**) and the SIX group compared with the ONE group (**J**). The enrichment takes padj < 0.05 as the threshold for significant enrichment.

Next, the effects of *A. muciniphila* on neighboring bacteria were explored through in vitro experiments. *L. murinus* drew our attention because of its high frequency and overt elevation following *A. muciniphila* treatment. Conditioned medium of *A. muciniphila* facilitated the proliferation of *L. murinus*, representing a faster growth rate and more bacterial colonies, which was consistent with the in vivo results (Fig. 3A and Supplementary Fig. 4a, b). Importantly, *L. murinus* alleviated DSS-induced colitis in mouse models (Supplementary Fig. 5). Thus, we chose *L. murinus* as the subject in the subsequent experiments, and the impacts of *A. muciniphila* on the gene expression profile of *L. murinus* were analyzed based on the coculturing time of the two anaerobic bacteria in vitro. Sterile cellophane allowed the exchange of metabolites while eliminating direct contact between the bacteria (Fig. 3B). Transcriptome sequencing showed that although both 1 h and 6 h cocultivation tuned the gene expression, 6 h cocultivation with *A. muciniphila* changed the transcriptome profile of *L. murinus* more obviously (Fig. 3C–F and Supplementary Fig. 4c). Coculturing with *A. muciniphila* for 1 h upregulated the expression of 456 genes and downregulated that of 502 genes in *L. murinus* (Fig. 3G). Compared with 1 h of cocultivation, 6 h of cocultivation further upregulated the expression of 563 genes and downregulated that of 558 genes (Fig. 3H), suggesting that *A. muciniphila* shifts the gene expression profiles of neighboring bacteria in a time-dependent manner. KEGG pathway enrichment analysis further showed that the pathways and associated genes related to ribosomal translation, such as the ribosome pathway and aminoacyl-tRNA biosynthesis (genes including *gat*, *DARS*, *TARS*, *VARS* and *HARS*), were enhanced following 1 h of cocultivation, while 6 h of cocultivation further enhanced the expression of these genes. Metabolic pathways such as glycolysis/gluconeogenesis (genes including *GPI*, *pgi1*, *pfk*, *PGK*, *ENO*, *PK* and *PKLR*) and carbon metabolism also changed in the same trend. Notably, the biosynthesis of some amino acids in *L. murinus* was enhanced following longer cocultivation with *A. muciniphila* only, including essential amino acids (leucine, isoleucine, valine and histidine) and nonessential amino acids (proline, arginine, alanine, methionine, tyrosine and phosphoserine) (Fig. 3I, J). GO functional analysis also revealed the same trend as the aforementioned analysis, especially for the amide biosynthetic process and cellular amide metabolic process (Supplementary Fig. 4d, e). Together, our observations demonstrate that *A. muciniphila* elicits proliferation and reprograms the gene expression profile of indigenous *L. murinus* in a time-dependent manner, hinting that monobacterial preparation optimization of the gut microecology relies on its retention and colonization in the GI tract.

### Increasing *A. muciniphila* colonization improves therapeutic efficacy in DSS-induced colitis

**Fig. 4:**
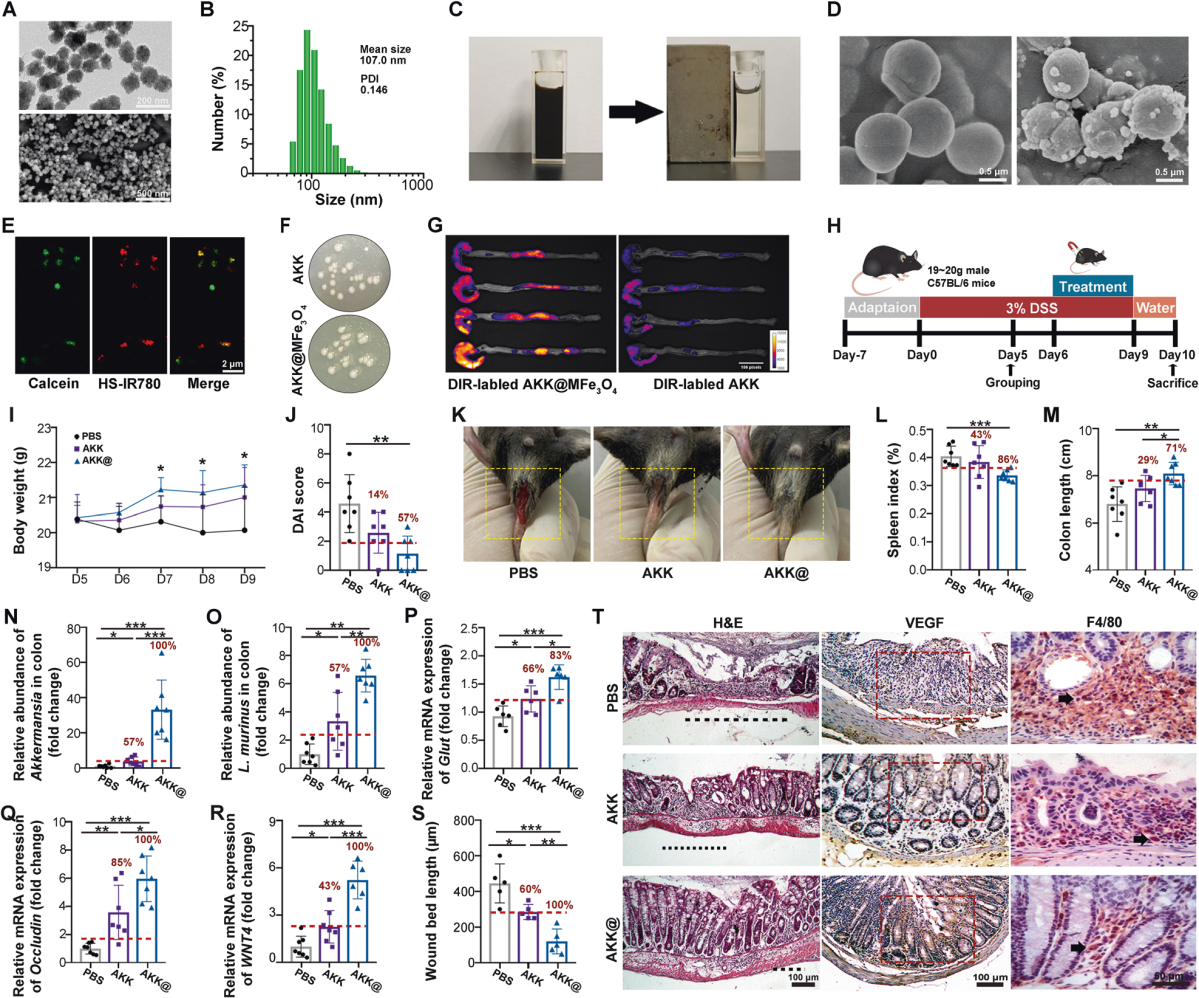
AKK@MFe_3_O_4_ improves the therapeutic efficacy against colitis. **A**, **B** TEM (top, scale bar: 200 μm), SEM (bottom, scale bar: 500 μm) images (**A**) and size distributions of MFe_3_O_4_ nanoclusters (**B**). **C** Magnetic attraction images of MFe_3_O_4_ nanoclusters. **D** SEM images of *A. muciniphila* (left) and AKK@MFe_3_O_4_ (right) (scar bar: 0.5 μm). **E** Colocalization fluorescence images of *A. muciniphila* and MFe_3_O_4_. *A. muciniphila* was stained with calcein (green), and MFe_3_O_4_ was labeled with HS-IR780 (red) (scar bar: 2 μm). **F** Clones of *A. muciniphila* and AKK@MFe_3_O_4_ that proliferated on plate medium. **G** Representative fluorescence images of colons derived from the mice treated with DIR-labeled AKK@MFe_3_O_4_ and DIR-labeled *A. muciniphila* with 3 h of magnetic attraction; colons were obtained immediately after the completion of magnetic attraction. **H** Scheme of the animal experimental design. Groups: PBS, the mice were administered PBS, *n* = 7; AKK, the mice were administered *A. muciniphila*, *n* = 7; AKK@, the mice were administered AKK@MFe_3_O_4_, *n* = 7. **I** Daily body weight of each mouse group until sacrifice. * indicates significant differences between the PBS and AKK@ groups. **J** The DAI score of colitis assessed on Day 10. **K** Representative photographs of rectal bleeding of mice after treatment with PBS, *A. muciniphila* and AKK@MFe_3_O_4_ (at Day 10). **L** Statistical results of the spleen index (the percentage of the weight of the spleen/body weight) of mice. **M** Statistical results of colon length. **N**, **O** The relative abundance of *A. muciniphila* (**N**) and *L. murinus* (**O**) in the colonic mucosa was detected by q-PCR. **P–R** The relative mRNA expression levels of *Glut, Occludin* and *Wnt4* were examined in colon tissues from mice by qRT‒PCR. **S** The wound bed lengths of the inflamed colon were measured (*n* = 5 wounds per group) with ImageJ. **T** Representative H&E images and IHC of VEGF and F4/80 proteins in the colon. The red box represents VEGF protein in the inflammation site (scar bar: 100 μm), and the black arrows point to macrophages (scar bar: 50 μm). **I**–**S** Significance was determined using one-way ANOVA corrected for multiple comparisons with an LSD *t*-test; *P < 0.05, **P < 0.01, ***P < 0.001. Data are the mean ± SD. The red numbers in the histogram indicate the percentage of positive responders in the *A. muciniphila* or AKK@MFe_3_O_4_ treatment group.

Next, we designed magnetic-drove, inflammation-targeted nanoparticles to prolong the retention time and improve the colonization of *A. muciniphila* in the colon. In light of macrophage targeting of mannose^36^, we assembled them with magnetic Fe_3_O_4_ nanoclusters (MFe_3_O_4_) to escalate the tendency toward inflammatory loci of the nanoparticles. Mannose alone showed no beneficial effect on IBD (Supplementary Fig. 6), and the morphology, size and magnetism of Fe_3_O_4_ nanoclusters were unaltered after mannose modification (Supplementary Fig. 7b, c and Fig. 4A–C). The changes in zeta potential and IR spectra were characterized to verify the modification of mannose (Supplementary Fig. 7d). The SEM and fluorescence colocalization images validated the successful linkage between *A. muciniphila* and nanoclusters (termed AKK@MFe_3_O_4_) (Fig. 4D, E). The MFe_3_O_4_ (100 μg/ml) linkage exhibited excellent magnetism in vitro (Supplementary Fig. 7e) and did not impact the proliferation and offspring morphology of *A. muciniphila* (Supplementary Fig. 7f, h and Fig. 4F). Furthermore, the magnetism and stability of AKK@MFe_3_O_4_ were retained by the freeze‒thaw process or in an acid-base environment (Supplementary Fig. 7i–l). The modified *A. muciniphila* presented a prolonged retention time in the GI tract under an external magnetic field (Fig. 4G).

Then, we evaluated the therapeutic effects of AKK@MFe_3_O_4_ (50 μg MFe_3_O_4_ and 5 × 10^7^ CFU of *A. muciniphila*/mouse) on colitis (Fig. 4H and Supplementary Fig. 8a). AKK@MFe_3_O_4_ treatment potentiated therapeutic efficacy in alleviating colitis compared to *A. muciniphila* (5 × 10^7^ CFU/mouse) under an external magnetic field, as judged by heavier body weight, less rectal bleeding, lower DAI score, lower spleen index and longer colon (Fig. 4I–M and Supplementary Fig. 8b, c). For positive response rate analysis, we calculated the proportion of mice with improvement based on the lack of therapeutic effects of PBS. In detail, the percentages of positive responders in the AKK@MFe_3_O_4_ group increased from 14 to 57% for the DAI score (Fig. 4J), 43–86% for the spleen index (Fig. 4I) and 29–71% for colon length compared to the AKK group (Fig. 4M). Notably, AKK@MFe_3_O_4_ increased the relative abundance of *A. muciniphila* by 35 times and promoted the proliferation of *L. murinus* in the colonic mucosa (Fig. 4N, O). AKK@MFe_3_O_4_ also ameliorated the colonic integrity of almost all experimental mice, while only approximately half of the mice responded to unmodified *A. muciniphila* (Fig. 4P, Q). In addition, AKK@MFe_3_O_4_ shortened the wound bed length, decreased the recruitment of macrophages to inflammatory sites, promoted the secretion of VEGF and upregulated the expression of *WNT4* compared to *A. muciniphila* alone (Fig. 4R–T). Together, our observations demonstrate that the magnetic-drove, inflammation-targeted *A. muciniphila* significantly improves the positive response rate and therapeutic efficacy to DSS-induced colitis in mouse models.

### AKK@MFe_3_O_4_ alleviates radiation-induced small intestinal injuries

**Fig. 5:**
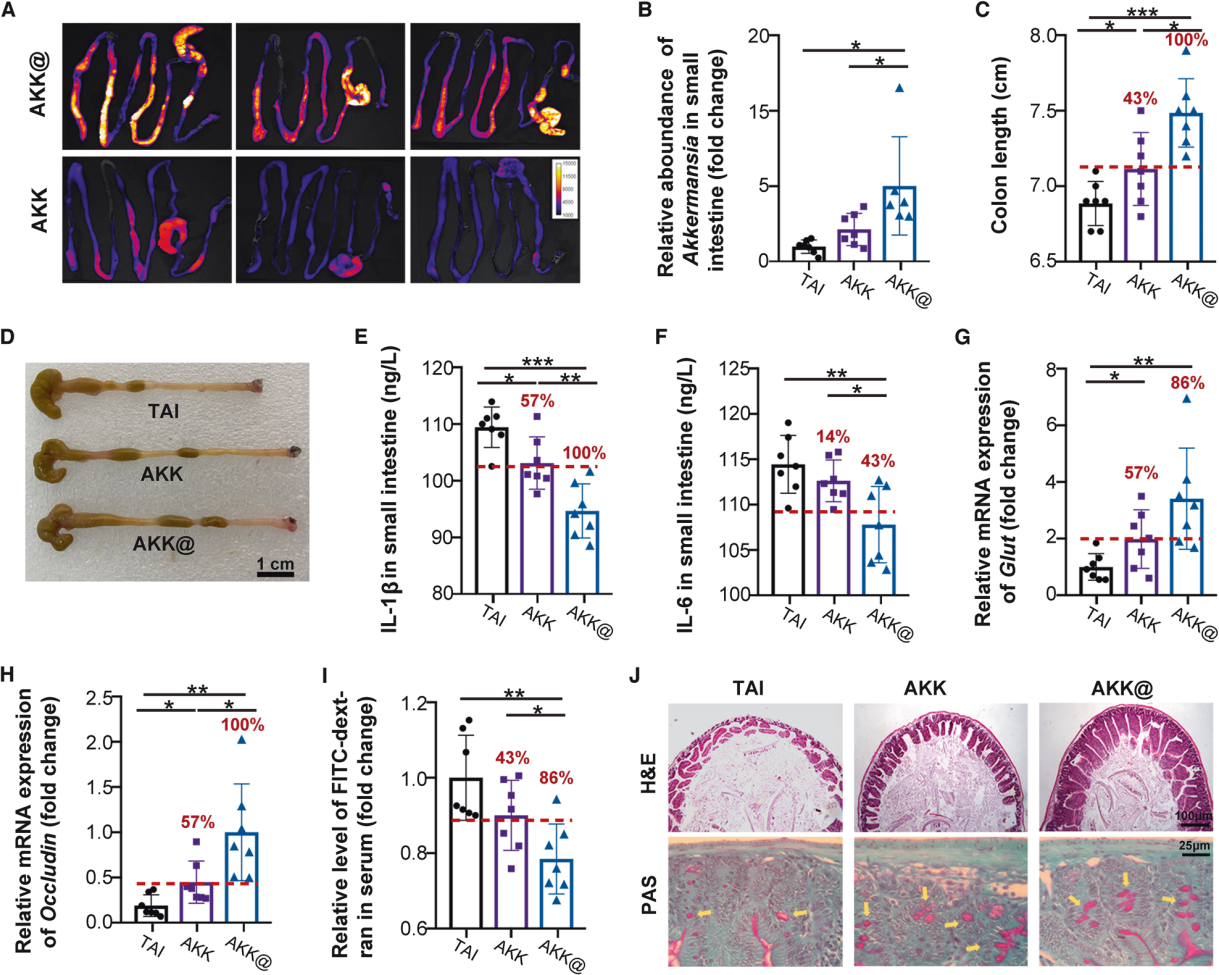
AKK@MFe_3_O_4_ alleviates radiation-induced small intestine injuries. All experimental mice received 12 Gy total abdominal irradiation (TAI), *n* = 7 per group. The red numbers in the histogram indicate the percentage of positive responders in each treatment group. **A** Fluorescence images of intestines derived from mice administered DIR-labeled AKK@MFe_3_O_4_ or *A. muciniphila* at 3 h following magnetic attraction completion. **B** The relative abundance of *A. muciniphila* in the small intestinal mucosa was detected by q-PCR. **C** Statistical results of colon length of mice with the indicated treatments at Day 21. **D** Representative photographs of the colon of mice at Day 21 (scar bar: 1 cm). **E**, **F** The levels of IL-1β and IL-6 in the small intestines of mice were measured by ELISA. **G**, **H** The relative mRNA expression levels of *Glut* and *Occludin* were examined in small intestines from mice by qRT‒PCR. **I** Fluorescence intensity of FITC-dextran in the serum of mice at Day 21. **J** Representative H&E images of intestinal villi (scar bar: 100 μm) and PAS staining of goblet cells in the small intestine (the yellow arrow points to the goblet cells, scar bar: 25 μm) are shown. **B**–**I** Significance was determined using one-way ANOVA corrected for multiple comparisons with an LSD *t*-test; *P < 0.05, **P < 0.01, ***P < 0.001. Data are the mean ± SD.

Although our previous study identified the protective effect of *A. muciniphila* against intestinal radiation injuries^26^, some individuals always showed an unsatisfactory response (Supplementary Fig. 9). Thus, we also evaluated the therapeutic efficacy of magnetic *A. muciniphila* on small intestinal radiation toxicities. As expected, AKK@MFe_3_O_4_ enhanced the retention and colonization of *A. muciniphila* in the small intestine (Fig. 5A, B), eliciting more positive responders with longer colons and lower inflammatory status in the small intestine (Fig. 5C–F). *A. muciniphila* alone improved the small intestinal integrity in ~50% of irradiated mice; however, AKK@MFe_3_O_4_ increased the response rate up to ~85% (Fig. 5G–I). In addition, AKK@MFe_3_O_4_ promoted the restoration of intestinal villi (Fig. 5J, the first line) and abrogated the loss of goblet cells following radiation (Fig. 5J, the second line). Together, our findings demonstrate that *A. muciniphila* retention and colonization are also essential for the therapeutic efficacy on radiation-induced small intestinal toxicity.

### A. muciniphila retention and colonization dictate the therapeutic efficacy of AKK@MFe_3_O_4_

**Fig. 6:**
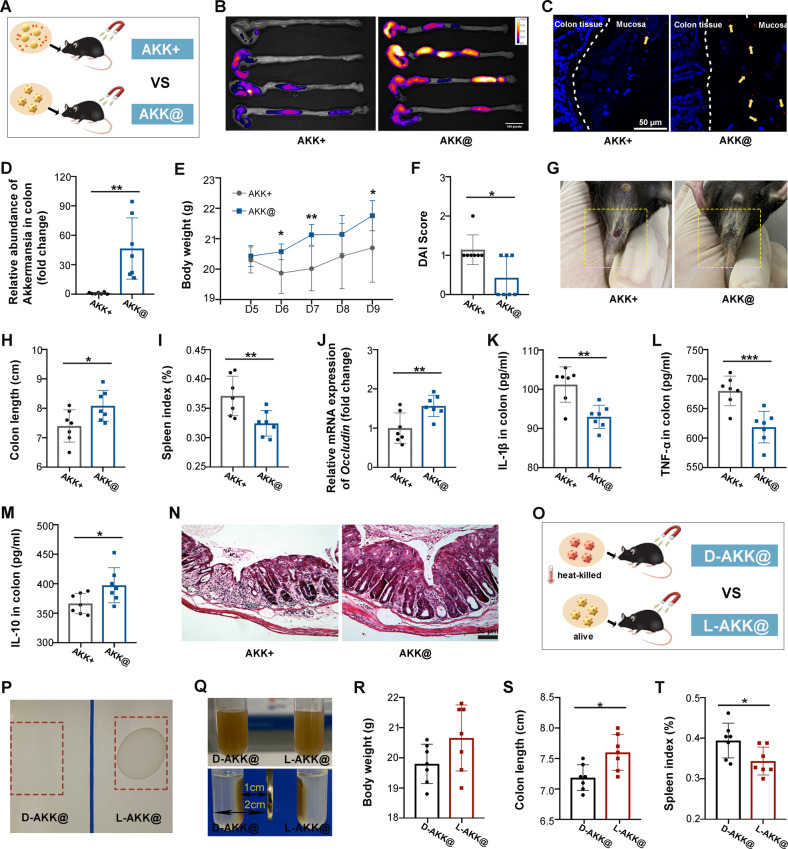
*A. muciniphila* colonization dictates the therapeutic efficacy of AKK@MFe_3_O_4_. **A** Scheme of the experimental design. AKK+, the mice were administered a mixture of MFe_3_O_4_ and *A. muciniphila*, *n* = 7; AKK@, the mice were administered magnetic *A. muciniphila* (AKK@MFe_3_O_4_), *n* = 7. **B** Fluorescence images of colons derived from mice administered DIR-labeled AKK@MFe_3_O_4_ or AKK + MFe_3_O_4_ at 3 h following magnetic attraction completion. **C** The relative abundance of *A. muciniphila* in the colonic mucosa was detected by q-PCR. **D** Confocal microscopy analysis of microbiota localization: bacteria, red and DNA, blue. The yellow arrow points to the bacteria (scar bar: 50 μm). **E** Daily body weight of each mouse group until sacrifice. **F** The DAI score of mouse colitis was assessed on Day 10. **G** Representative photographs of rectal bleeding of mice on Day 10. **H** Statistical results of colon length of mice. **I** Statistical results of the spleen index of mice. **J** The relative mRNA expression levels of *Occludin* were examined in colon tissue from mice by qRT‒PCR. **K–M** The levels of IL-1β, TNF-α and IL-10 in the inflamed colons of mice were measured by ELISA. **N** Representative H&E images of the colon are shown (scale bar: 50 μm). **O** Scheme of the experimental design. D-AKK@, the mice were administered AKK@MFe_3_O_4_ of heat-killed *A. muciniphila*, *n* = 7; L-AKK@, the mice were administered AKK@MFe_3_O_4_ of live *A. muciniphila*, *n* = 7. **P** Colony formation of AKK@MFe_3_O_4_ with dead or live *A. muciniphila*. **Q** The magnetic attraction images of AKK@MFe_3_O_4_ with dead or live *A. muciniphila* in vitro. **R** The body weight of mice at Day 10. **S** Statistical analysis of colon length. **T** Statistical results of the spleen index. Significance was determined using Student’s *t test*; *P < 0.05, **P < 0.01, ***P < 0.001. Data are the mean ± SD.

To identify the role of *A. muciniphila* colonization in elevating the therapeutic efficacy of AKK@MFe_3_O_4_, we mixed *A. muciniphila* and MFe_3_O_4_ together without chemical linkages (termed AKK + MFe_3_O_4_, 50 μg MFe_3_O_4_ and 5 × 10^7^ CFU of *A. muciniphila*), which could not detain *A. muciniphila* in the GI tract under magnetic attraction (Supplementary Fig. 10). As shown in Supplementary Fig. 11, although the mixture showed better therapeutic effects than *A. muciniphila* or MFe_3_O_4_ alone, no obvious additive or synergistic effect of the mixture was observed, indicating that *A. muciniphila* colonization in the GI tract might play more important roles in fighting against colitis. Then, the therapeutic effects of AKK@MFe_3_O_4_ and AKK + MFe_3_O_4_ were further compared (Fig. 6A). In vivo imaging analysis showed that the MFe_3_O_4_ linkage spurred stronger retention of *A. muciniphila* than AKK + MFe_3_O_4_ (Fig. 6B). qPCR revealed a nearly 40-fold elevation in *A. muciniphila* abundance in the colon mucosa of mice in the AKK@MFe_3_O_4_ group compared with the AKK + MFe_3_O_4_ group after administration (Fig. 6C, D). As expected, mice with DSS-induced colitis experienced better therapeutic effects with AKK@MFe_3_O_4_ treatment compared to AKK + MFe_3_O_4_, as judged by more individuals exhibiting lower body weight and DAI score, less serious rectal bleeding, longer colon and lower spleen index (Fig. 6E–I and Supplementary Fig. 12a). In addition, more mice carried higher expression of integrity genes and lower inflammatory status in colon tissues in the AKK@MFe_3_O_4_ group (Fig. 6J–M and Supplementary Fig. 12b). H&E staining further revealed that AKK@MFe_3_O_4_-treated mice possessed less impaired colon structure than their AKK + MFe_3_O_4_-treated counterparts (Fig. 6N). We also inactivated *A. muciniphila* at 100 °C for 15 min. Then, the heat-killed *A. muciniphila* was modified by MFe_3_O_4_ and administered orally to the mice with colitis (Fig. 6O–Q). The results showed that heat-killed *A. muciniphila* lost the therapeutic effects (Fig. 6R–T and Supplementary Fig. 12c, d). Together, our observations demonstrate that retention and colonization of live *A. muciniphila* contribute to the high positive-response rate and therapeutic efficacy of AKK@MFe_3_O_4_.

### AKK@MFe_3_O_4_ facilitates M2 macrophage polarization in the colon of mice with DSS-induced colitis

**Fig. 7:**
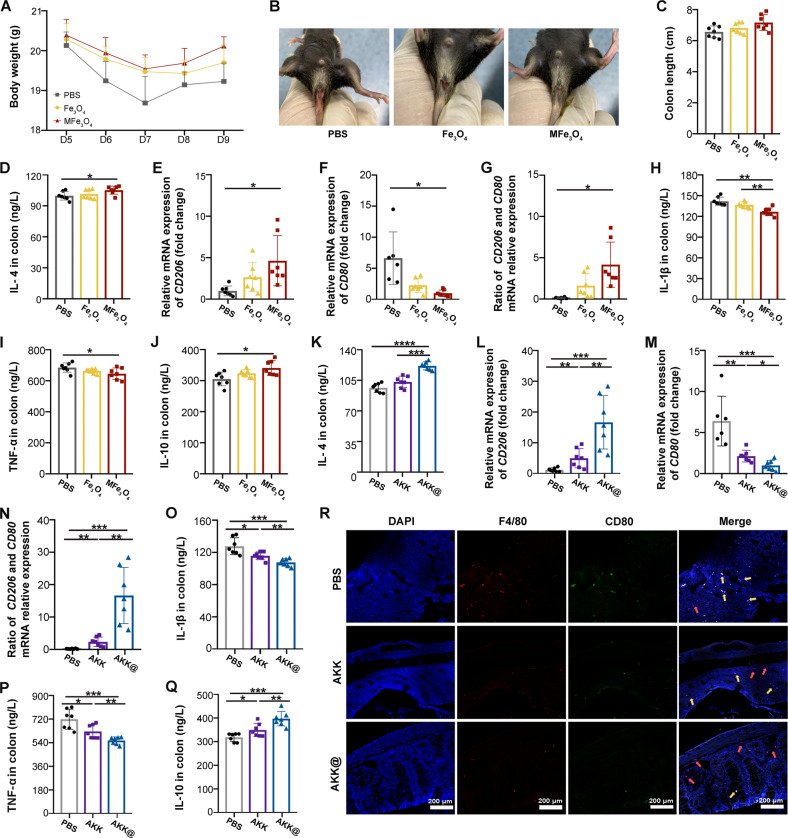
AKK@MFe_3_O_4_ facilitates M2 macrophage polarization in the colon of mice with colitis. Groups: PBS, the mice were administered PBS, *n* = 7; Fe_3_O_4_, the mice were administered Fe_3_O_4_; MFe_3_O_4_, the mice were administered MFe_3_O_4_; AKK, the mice were administered *A. muciniphila*; AKK@, the mice were administered AKK@MFe_3_O_4_. **A** Daily body weight of each mouse group until sacrifice. **B** Representative photographs of rectal bleeding of IBD mice at Day 10. **C** Statistical results of colon length of mice. **D** The levels of IL-4 in inflamed colon homogenate of mice were measured by ELISA. **E**, **F** The relative mRNA expression levels of *CD206* and *CD80* were examined in colon tissues from mice by qRT‒PCR. **G** The ratio of mRNA expression levels of *CD206* and *CD80*. **H–J** The levels of IL-1β, TNF-α and IL-10 in inflamed colon homogenate of mice were measured by ELISA. **K** The levels of IL-4 in inflamed colon homogenate of mice in the PBS, AKK and AKK@ groups were measured by ELISA. **L**, **M** The relative mRNA expression levels of *CD206* and *CD80* were examined in colon tissue from mice in the three groups measured by qRT‒PCR. **N** The ratio of mRNA expression levels of *CD206* and *CD80*. **O–Q** The levels of IL-1β, TNF-α and IL-10 in inflamed colon homogenate of mice in the three groups measured by ELISA. **R** Representative IHC of total macrophages and M1 macrophages in the colon. Total macrophages, F4/80, red; M1 macrophages, CD 80, green; and DNA, DAPI, blue. The yellow and red arrows point to M1 and M2 macrophages, respectively (scale bar: 200 μm). Significance was determined using one-way ANOVA corrected for multiple comparisons with an LSD-t test; *P < 0.05, **P < 0.01, ***P < 0.001. Data are the mean ± SD.

Finally, we investigated the underlying mechanism of the *A. muciniphila-*based therapeutic system. The effects of Fe_3_O_4_ (50 μg/mouse) and MFe_3_O_4_ (50 μg/mouse) on colitis were investigated first. Oral gavage of MFe_3_O_4_ exhibited better therapeutic efficacy against colitis than Fe_3_O_4_, as judged by the increase in body weight and colon length as well as the decrease in the DAI score and spleen index (Fig. 7A–C and Supplementary Fig. 13a, b). Histological analysis also showed more obvious improvement of MFe_3_O_4_ on colitis (Supplementary Fig. 13c)_._ Given the close relationship between macrophages and intestinal inflammation^37^, we found that oral gavage of MFe_3_O_4_ with external magnetic force increased the content of IL-4, a key modulator of macrophages^38^, in colon tissues from mice with DSS-induced colitis (Fig. 7D). Compared to Fe_3_O_4_, MFe_3_O_4_ also showed more obvious upregulation of M2 macrophage marker expression (*CD206*, Fig. 7E) and downregulation of that of M1 macrophages (*CD80*, Fig. 7F), as well as sharper elevation in the CD206/CD80 ratio (Fig. 7G). The changes in the contents of M1 macrophage-secreted IL-1β and TNF-α and M2 macrophage-secreted IL-10 further validated the functions of Fe_3_O_4_ and MFe_3_O_4_ (Fig. 7H–J), suggesting that the magnetic nanoparticles elicit the conversion of macrophages from the proinflammatory type (M1) to the inflammation-inhibiting type (M2) in vivo. Importantly, although *A. muciniphila* alone exhibited significant macrophage polarization, AKK@MFe_3_O_4_ administration with external magnetic force accumulated a higher proportion of M2-polarized macrophages, as judged by the elevation of the marker and secretion of M2 macrophages and the reduction of those of M1 macrophages (Fig. 7K–Q). Immunofluorescence assays further revealed the disappearance of M1-polarized macrophages following AKK@MFe_3_O_4_ treatment (Fig. 7R). Together, our observations indicate that AKK@MFe_3_O_4_ exerts anti-inflammatory effects by driving M2 macrophage polarization at least partly.

## Discussion

Dysbiosis in gut microbiota is closely related to the occurrence and development of multiple diseases^39^. Although therapeutic strategies targeting the gut microbiome, such as FMT and diverse probiotics, have been progressively put into use in clinical trials^40–42^, the curative effects have yielded mixed reception^20^. For example, *Bifidobacterium bifidum* as an adjuvant preparation to potentiate immunity therapy for lung cancer was obliged to oral gavage every day to surmount the low response rate^22^. In line with the outcomes from clinical trials of probiotic or probiotic mixtures such as *Vivomixx*^43^, we also observed a considerable proportion of poor responding recipients (approximately more than 50%) when *A. muciniphila* was used to alleviate colitis in mouse models and further found that the escape of orally gavaged *A. muciniphila* from the intestine might be a key reason for the mixed success of the treatment. Enhancing the colonization of *A. muciniphila* in the GI tract not only elevated the response rate from ~50% to almost 100% but also improved the curative efficacy against colitis and intestinal radiation toxicity. Notably, the evaluating indicators for intestinal injuries responding to *A. muciniphila* treatment poorly showed only finite improvement even under the condition of stable colonization. In our opinion, these results might stem from the physiological characteristics of *A. muciniphila* itself, which requires further study. However, as a monobacterial preparation, why does the colonization of *A. muciniphila* in the GI tract impact its therapeutic efficiency?

Bacteria, archaea, viruses and fungi dwelling in the GI tract communicate with each other, forming a sophisticated and dynamic microecosystem with the host^44^. In our study, qPCR and 16 S rRNA sequencing showed that the education on gut microecology by *A. muciniphila* depended on its stable colonization. Gut microbiota-derived metabolites and cellular components are regarded as key modulators and remain the research hotspots in this field^45^. Studies report that pasteurized *A. muciniphila* or a specific outer membrane protein from *A. muciniphila* (Amuc_1100) is enough to alleviate metabolic disease or colitis^32^. However, we found that heat-killed *A. muciniphila* lost its anti-inflammatory effects on colitis in mouse models. Live *A. muciniphila* with biological activity exhibits advantages in secreting beneficial metabolites and interacting with neighboring microbes^46,47^. Thus, we used conditioned medium and a coculture system to further evaluate the effects of *A. muciniphila* on other intestinal bacteria. *L. murinus* is a potential probiotic with anti-inflammatory activity^48^ and showed an accumulation in the GI tract of *A. muciniphila*-responsive mice in our study. The coculture system in our study avoided direct contact between the two bacteria (details shown in the methods). Intriguingly, *A. muciniphila* facilitated proliferation and reprogrammed the gene expression profile of *L. murinus via* its derived metabolites. Six hours of coexistence with *A. muciniphila* further activated biological processes and signaling pathways related to amino acid metabolism in *L. murinus*. Amino acids regulate intestinal health, and some amino acids are metabolized by gut microbiota to produce numerous secondary metabolites, such as branched-chain amino acids and indolic compounds, which are beneficial for intestinal homeostasis^49^. Some metabolic pathways related to SCFA production, such as carbon metabolism, glycolysis and pyruvate metabolism, were upregulated in *L. murinus* following coexisting with *A. muciniphila*, which might further promote the recovery of intestinal inflammation^50^. Owing to the crosstalk among gut microbes, the application of live probiotics with colonization ability might be a suitable option to result in sustained and effective therapeutic effects. However, there are some limitations in our experimental system. First, the coculture system was performed in vitro, and the impacts of *A. muciniphila* on the gene expression of *L. murinus* in vivo should be validated in future studies. Second, the experimental strategy cannot fully represent the complex gut microbiome environment due to the neglect of the influences of direct contact between bacteria, etc. Finally, other bacteria regulated by *A. muciniphila* colonization might also play important roles and remain to be further explored. Despite these findings, our results provide some reference value for the regulation mode of *A. muciniphila* on microbes with spatial separation.

There are many different delivery strategies to promote probiotic colonization in the GI tract. Hydrogel and biofilm-based delivery systems are able to assist probiotics in staying in the GI tract stably^51,52^; however, these strategies have difficulty controlling the duration and location of probiotic colonization accurately. In broad terms, magnetic materials modulated by external magnetic force have emerged as superior carriers for probiotics to battle against GI diseases^53^. Driven by external magnetic fields, magnetic bacteria can achieve directional accumulation in the GI tract and long-term colonization^54^. Here, we designed a two-target magnetic delivery system to promote the precise colonization of *A. muciniphila* at GI lesion sites. On the one hand, Fe_3_O_4_ nanoparticles maintained magnetic targeting and elicited M2 macrophage polarization. On the other hand, mannose had no effect on colitis but guided Fe_3_O_4_ to target the inflammatory locus. Importantly, the novel nanoparticles did not impact the proliferation of *A. muciniphila* and *L. murinus* (Supplementary Fig. 7f, g). Compared to the traditional single-target magnetic system, our delivery materials were capable of performing macropositioning of combined probiotics in the GI tract under an external magnetic field. Furthermore, macrophage-targeted mannose potentiated inflammatory locus trends to elicit micropositioning of probiotics. As a result, the two-target magnetic system increased *A. muciniphila* by ~40-fold during colon colonization and 5-fold during small intestinal colonization, improving the response rate and therapeutic efficacy. Although some studies reported the potential side effects of iron overload^55^, our results showed that 50 μg Fe_3_O_4_ or MFe_3_O_4_ per mouse was beneficial for the alleviation of DSS-induced colitis in mouse models. In our opinion, the differences in valence and states of iron (such as oxidation state, polymerization state, particle size and potential, etc.) might affect the absorption process of iron in the intestine^56^. In addition, in vitro TEM images and in vivo imaging indicated that MFe_3_O_4_ was diluted following the proliferation of *A. muciniphila* and finally excreted out of the GI tract with the feces (Supplementary Figs. 7h, 14). MFe_3_O_4_ combined with *A. muciniphila* was stable in different acid-alkali environments. These signatures might guarantee the safety of the magnetic delivery system in the digestive tract. A study reported that *A. muciniphila* alleviates DSS-induced acute colitis by *NLRP3* activation^57^. Overexpression of *NLRP3* elevates IL-4 secretion to reduce the M1/M2 macrophage ratio^58^, implying that *A. muciniphila* has the potential to facilitate M2 macrophage polarization. Here, we identified that *A. muciniphila* indeed increased the level of IL-4, resulting in M2 macrophage polarization in the colitis locus. In addition, MFe_3_O_4_ exhibited more efficacious effects on macrophage polarization than pure Fe_3_O_4_ nanoparticles owing to the macrophage targeting of mannose. Overall, although MFe_3_O_4_ and *A. muciniphila* showed a certain extent of macrophage polarization, *A. muciniphila* and MFe_3_O_4_ linkage performed much stronger M2-polarized function and therapeutic efficacy than *A. muciniphila* or MFe_3_O_4_ alone as well as *A. muciniphila* and MFe_3_O_4_ mixture without linkage. This further suggests that the colonization of *A. muciniphila* at the lesion site is critical for its treatment of inflammatory intestinal diseases. However, there are still some limitations and challenges in this study. For example, the optimal dose and state of iron nanoparticles need to be further screened to avoid excess iron entering the circulation system. In addition, more magnetic materials with appropriate magnetic force, biocompatibility and biosafety are promising to explore.

**Fig. 8:**
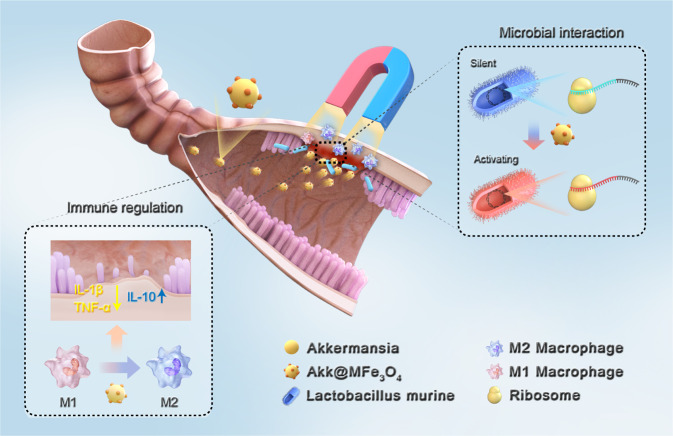
Schematic diagram of the function of AKK@MFe_3_O_4_. Magnetic delivery system (MFe_3_O_4_) enhances the colonization of *A. muciniphila* in inflammatory sites, alleviating inflammation by regulating host intestinal immunity and microecology.

In summary, our findings indicate that the mixed success of *A. muciniphila* in colitis and irradiation intestinal injury treatment is related to colonization failure in the GI tract. Stable colonization of *A. muciniphila* prompts proliferation and modulates the gene expression profiles of neighboring bacteria. A magnetic and inflammatory targeting delivery system is designed to potentiate the retention and colonization of *A. muciniphila* in the GI tract. Magnetic *A. muciniphila* facilitates M2 macrophage polarization and increases the response rate and therapeutic efficacy to colitis and irradiation intestinal injury (Fig. 8). Clinically, mannose combined with magnetic nanoparticles is an optimized option and might be employed as a safe and effective delivery system to surmount colonization failure of microbial-based therapy for inflammatory GI diseases.

## Supplementary information

Supplementary materials

The movement of mice wearing magnetic device

## Data Availability

The sequencing data have been deposited to the European Nucleotide Archive with the dataset identifiers PRJEB47413, and the other data have all been presented in the paper. All other data supporting the findings of this study are available from the corresponding author upon reasonable request.
