# Supplementary material for: Stable colonization of Akkermansia muciniphila educates host intestinal microecology and immunity to battle against inflammatory intestinal diseases
Source: Exp Mol Med. 2023 Jan 4;55(1):55–68. doi: 10.1038/s12276-022-00911-z (PMC9898499; doi:10.1038/s12276-022-00911-z)
Supplement: Supplementary file 1 — Supplementary materials [file 12276_2022_911_MOESM1_ESM.pdf]

Supplementary Materials

Supplementary methods

GI dynamics of bacteria

In order to visualize the dynamics of *A. muciniphila* in the GI tract of DSS mice, fluorescent labelling for *A. muciniphila* was performed firstly. Briefly, *A. muciniphila* (OD_{600} = 0.8) suspended in 1 ml anaerobic PBS was co-incubated with 20 μM DIR (dissolved in 5 μl DMSO) at 37 °C for 1 h. The labelled bacteria were washed by anaerobic PBS three times to remove residual DIR and resuspended with anaerobic PBS following centrifugation (3000 rpm, 5 min). DIR labelled *A. muciniphila* was administrated orally to mice with DSS-induced acute colitis at the concentration of 1×10^8 CFU/200 μl per mouse. Then, the mice were sacrificed and the intestines were rapidly removed at 0.5 h, 1 h, 1.5 h, 2 h, 3 h and 5 h respectively (three mice at each time point) and stored at 4 °C in dark. *Ex vivo* imaging was used for detecting the *A. muciniphila* in dissected intestines (In-Vivo Master at 635-690 nm with the same exposure time-0.3 s). Data acquisition and analyses were done with built-in software.

Clone formation

A. muciniphila (1×10^5 CFU) from a preculture was incubated in 20 ml liquid BHI medium for 6 h followed by centrifuging at 10000 g for 10 min, and the supernatant was passed through a 0.22 μm filter membrane for use. Add fresh liquid BHI medium or supernatant (CM-medium, 100 μl) to the MRS solid medium respectively. *L. murinus* suspension (OD_{600}=0.4) was diluted 10,000 times by anaerobic PBS and then inoculated into the medium with different pre-

treatment respectively. After culturing for 24 h under strict anaerobic incubator (90% N_2 and 10% H_2), the number of clones was counted.

***L. murinus* experiments**

For growth rate, fresh liquid BHI medium or CM-medium (50 μl) was added to the MRS liquid medium (1 ml) respectively. Then *L. murinus* (OD_{600} = 0.4, 10 μl) was inoculated into the pre-treated medium. The OD_{600} value was detected at different time points following culturing under strict anaerobic incubator (90% N_2 and 10% H_2).

To explore the effects of *L. murinus* on acute colitis, the mice with DSS-induced acute colitis were administrated orally with *L. murinus* (1×10^8 CFU/200 μl per mouse) for 3 days while the contrast mice were given sterile PBS with an equivalent volume.

Preparation and characterization of MFe_3O_4

490 mg $FeCl_3 \cdot 6H_2O$ was firstly dissolved in ethylene glycol (20 ml), then 200 μL trisodium citrate dihydrate solution (200 mg) and 1.2 g sodium acetate were added with stirring. After further stirred for 30 min, the mixture was sealed into a Teflon-lined stainless-steel autoclave (50 ml capacity). Then the autoclave was heated to 200 ℃ and maintained for 6 h. Finally, the products were obtained after washed with deionized water for three times under magnet attraction. To prepare the mannose decorated Fe_3O_4 nanoclusters (termed as MFe_3O_4), D-mannosamine hydrochloride (20 mg, 92.6 μM) and Traut's reagent (13 mg, 92.6 μM) were firstly reacted in 1 ml ultra-dry DMSO with triethylamine (139 μM) as dehydrochloride reagent for 4 h to obtain the sulfhydryl mannose. Then 100 mg Fe_3O_4 nanoclusters dispersing in 50 ml deionized water were added into above mixture and further shaken at 37 ℃ for another 4 h to

allow sulfhydryl mannose to chelate onto Fe_3O_4 nanoclusters. The obtained MFe_3O_4 was finally acquired after washed with deionized water. The morphology and hydrodynamic diameter, and zeta potentials of Fe_3O_4 and MFe_3O_4 nanoclusters were characterized by transmission electron microscope (TEM), scanning electron microscope (SEM) and dynamic laser scattering (DLS) respectively. The magnetic property of MFe_3O_4 was evaluated through a rubidium magnet. Besides, the IR spectra of Fe_3O_4, D-mannosamine hydrochloride and MFe_3O_4 were characterized by a Fourier transform infrared spectrometer to check the mannose coupling.

Toxicity assessment of MFe_3O_4

Before preparing AKK@MFe_3O_4, the toxicity of MFe_3O_4 on AKK was firstly evaluated. *A. muciniphila* (1×10^8 CFU) was co-incubated with free MFe_3O_4 of different concentration (0, 50, 100, 150 and 200 μg/ml on the basis of Fe_3O_4) in 1 ml anaerobic PBS for 6 h followed by centrifuging at 3000 rpm for 5 min. After resuspended with 1 ml anaerobic PBS, 10 μl resuspension was inoculated into 1 ml liquid BHI for 12 h. Then the precipitate agglomerate of *A. muciniphila* was observed. All experiments were under anaerobic conditions. For *L. murinus*, *L. murinus* (1×10^8 CFU) was co-incubated with free MFe_3O_4 of different concentration (0, 50, 100 μg/ml on the basis of Fe_3O_4) for 6 h. Finally, 10 μl resuspension (OD_{600} = 0.4) was inoculated into 1 ml liquid MRS for 24 h. Then the precipitate agglomerate and OD_{600} value were assessed.

Measurement of connection efficiency

A standard curve of free MFe_3O_4 nanoparticles versus absorbance was first established using UV spectroscopy at 300 nm. When the reaction completed, the obtained AKK@ MFe_3O_4 was

separated (3000 rpm, 5 min) and the supernatant was used to detect the amount of unlinked MFe_3O_4. The connection efficiency is calculated to be 95.14%.

Fluorescence co-localization

Firstly, the IR 780 labelled MFe_3O_4 was prepared by the co-incubation of HS-IR780 (3 mg) with MFe_3O_4 (5 mg) in 2.5 ml deionized water under 100 rpm shaking for 4 h. The success of connections was confirmed by UV Spectrum at 785 nm. Then MFe_3O_4 labelled by IR780 was used to prepare AKK@MFe_3O_4 as described above method described. The obtained AKK@MFe_3O_4 was further stained with calcein-AM (a living cell dye) for 1 h followed by imaging *via* confocal microscope to observe the co-localization of MFe_3O_4 and *A. muciniphila*.

Mannose treatment

To explore the effects of mannose (aladdin, Shanghai, China) on acute colitis, the mice with DSS-induced acute colitis were administrated orally with mannose of experimental concentration (10 μg/per mouse, dissolved in PBS) for 3 days while the contrast mice were given PBS with an equivalent volume.

Histology

For hematoxylin and eosin (H&E) staining, the intestine tissues were fixed in 4% buffered formalin and then horizontally embedded in paraffin. Tissues were sectioned at 5 μm thickness and performed H&E staining according to standard protocols. The crypt structures of colon or small intestinal villi were pictured, and the wound bed lengths of colons were measured and statistically analyzed using ImageJ. For PAS staining, the small intestines of mice were fixed in Carnoy's solution (absolute ethanol: chloroform: glacial acetic acid = 6:3:1) for 12 h.

Dewaxed sections were hydrated and incubated in 1% periodic acid for 10 min followed by incubation in Schiff's reagent for 10 min. Sections were counterstained with Mayer's hematoxylin for 30 s, washed and dehydrated before mounting with Pertex. For immunohistochemical staining (IHC) of colon tissues, the primary antibody of rat anti-F4/80 (Santa Cruz Biotechnology, USA) and VEGF (Proteintech Group, Chicago, USA) were used. Sections were examined under light microscopy and categorization of immunostaining intensity was performed by three independent researchers. As for immunofluorescence staining of macrophages in colon tissues, rat anti-F4/80 (Santa Cruz Biotechnology, USA, diluted to 1:500 in PBS), rabbit anti-CD80 (Santa Cruz Biotechnology, USA, diluted to 1:500 in PBS), mouse Alexa 647 secondary antibody (Abcam, diluted to 1:1000 in PBS) and rabbit Alexa 488 secondary antibody (Abcam, diluted to 1:1000 in PBS) were applied to immunostaining of total macrophages and M1 type macrophages. Finally, slides were mounted using prolong anti-fade mounting media containing DAPI (Biosharp) and the observations were performed with Leica microsystems.

Bacterial diversity analysis

DNA was extracted from the stool using the Power Fecal® DNA Isolation Kit (MoBio, Carlsbad, CA USA). The DNA was recovered with 30 ml of buffer in the kit. PCR products were mixed in equidensity ratios. Then, the mixture was purified with Qiagen Gel Extraction Kit (Qiagen, Hilden, Germany). The 16S ribosomal RNA (rRNA) V4 gene was analyzed to evaluate the bacterial diversity using lonS5TMXL lon 530 Chip (Thermo fisher, Waltham, MA, USA). Sequences analysis was performed by Uparse software (Uparse v7.0.1001,

http://drive5.com/uparse/). Sequences with ≥ 97% similarity were assigned to the same OTUs. Representative sequence for each OTU was screened for further annotation. For each representative sequence, the Silva123 Database was used based on RDP classifier (Version 2.2, http://sourceforge.net/projects/rdpclassifier/) algorithmto annotate taxonomic information. For TOP10 and single bacteria abundance analysis, extreme values were not included in the analysis.

Transcriptome sequencing

For Library preparation for Transcriptome sequencing, total RNA was used as input material for the RNA sample preparations. For prokaryotic samples, mRNA was purified from total RNA using probes to remove rRNA. Fragmentation was carried out using divalent cations under elevated temperature in First Strand Synthesis Reaction Buffer (5X). First strand cDNA was synthesized using random hexamer primer and M-MuLV Reverse Transcriptase, then use RNaseH to degrade the RNA. And in the DNA polymerase I system, use dUTP to replace the dNTP of dTTP as the raw material to synthesize the second strand of cDNA. Remaining overhangs were converted into blunt ends via exonuclease/polymerase activities. After adenylation of 3’ ends of DNA fragments, Adaptor with hairpin loop structure were ligated to prepare for hybridization. Then USER Enzyme was used to degrade the second strand of cDNA containing U, in order to select cDNA fragments of preferentially 370~420 bp in length, the library fragments were purified with AMPure XP system (Beckman Coulter, Beverly, USA). Then PCR was performed with Phusion High-Fidelity DNA polymerase, Universal PCR primers and Index (X) Primer. At last, PCR products were purified (AMPure XP system) and library quality was assessed on the Agilent Bioanalyzer 2100 system. For Clustering and

sequencing (Novogene Experimental Department), the clustering of the index-coded samples was performed on a cBot Cluster Generation System using TruSeq PE Cluster Kit v3-cBot-HS (Illumia) according to the manufacturer's instructions. After cluster generation, the library preparations were sequenced on an Illumina Novaseq platform and 150 bp paired-end reads were generated. In the data analysis stage, quality control, reads mapping to the reference genome, novel gene and gene structure analyzing, quantification of gene expression level, differential expression analysis and GO and KEGG enrichment analysis of differentially expressed genes were conducted.

Supplementary tables

Supplementary Table 1 Scoring system for division of PR and R mice.

**Score**				
**(IBD)**	**DAI**			**Colon**
	Weight loss	Stool consistency	Blood	Length
0	None	Normal	Negative hemocult	8.5-9 cm
1	1-5%	Soft but still formed	Positive hemocult	8-8.5 cm
2	6-10%	Very soft; wet	Blood traces in stool visible	7.5-8 cm
3	11-18%	Watery diarrhea	Gross rectal bleeding	7-7.5 cm
4	>18%			6.5-7 cm
**(IRD)**				
0				8.5-9 cm
1				8-8.5 cm
2				7.5-8 cm

3				7-7.5 cm

IBD: inflammatory bowel disease; IRD: intestinal radiation disease.

Supplementary Table 2 Primers for PCR and sequencing.

**Gene**	**Primer**	**Sequence (5′-3′)**
**Primers for qRT-PCR**		
GAPDH	forward	TGTTTCCTCGTCCCGTAGA
	reverse	CAATCTCCACTTTGCCACTG
Glut	forward	TATCCTGTTGCCCTTCTGC
	reverse	CCGACCCTCTTCTTTCATCTC
Occludin	forward	GCCTTCCTGGACCACAACA
	reverse	CCGTCGGATCATAGAACTCG
WNT4	forward	GAGAAGTTTGACGGTGCC
	reverse	GCCGTCAATGGCTTTAG
CD206	forward	CATTCCCTCAGCAAGCGATG
	reverse	GGGTTCCATCACTCCACTCA
CD80	forward	TTCGTCTTTCACAAGTGTCTTCA
	reverse	TGCCAGTAGATTCGGTCTTCA
**Primers for sequencing**		
515F		GTGCCAGCMGCCGCGGTAA
806R		GGACTACHVGGGTWTCTAAT
**16S rRNA Primers for q-PCR**		
Bacterial universal	forward	ACTCCTACGGGAGGCAGCAG
	reverse	ATTACCGCGGCTGCTGG
A. muciniphila	forward	CAGCACGTGAAGGTGGGGAC
	reverse	CCTTGCGGTTGGCTTCAGAT
L. murinus	forward	ACTGGCGATGTTACCTTTGG
	reverse	CAGGCCTTGTATTGGTGGT

Supplementary Table 3 Notes for the numbers in Fig. 2j.

**01**	**Actinobacteria**	**02**	**Synergistia**	**03**	**Caldatribacteriia**	**04**	**Anaerolineae**
**05**	**Kiritimatiellae**	**06**	**Aminicenantia**	**07**	**Deferribacteres**	**08**	**Cyabobacteriia**
**09**	**Saccharimonadia**	**10**	**Nanosalinia**	**11**	**Halobacteria**	**12**	**Desulfovibrionia**
**13**	**Coprothermobacteria**	**14**	**Coriobacteriia**	**15**	**Bacteroidia**	**16**	**Spirochaetia**
**17**	**Fibrobacteria**	**18**	**Bacteroidia**	**19**	**Vampirivibrionia**	**20**	**Clostridia**
**21**	**Campylobacteria**	**22**	**Methanomicrobia**	**23**	**Clostridia**	**24**	**Alphaproteobacteria**
**25**	**Desulfovibrionia**	**26**	**Bacilli**	**27**	**Negativicutes**	**28**	**D8A-2**
**29**	**Bacilli**	**30**	**Coriobacteriia**	**31**	**Methanobacteria**	**32**	**Fusobacteriia**
**33**	**Gammaproteobacteria**	**34**	**Verrucomicrobiae**	**35**	**Alphaproteobacteria**		

Supplementary figures

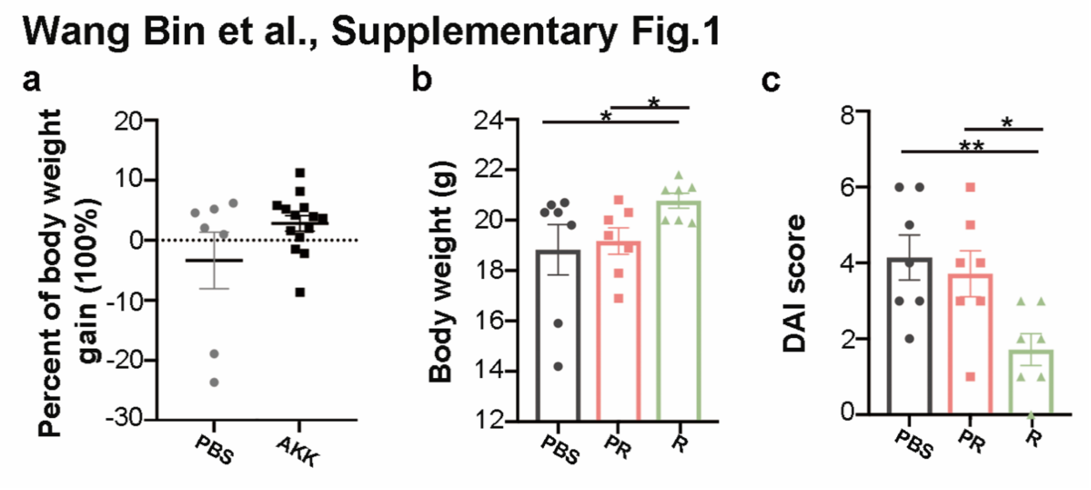

Supplementary Fig. 1 ***A. muciniphila*** **colonization correlates with its therapeutic efficacy to DSS-induced colitis.**

(a) The percent of body weight gain at day 10. **(b)** The body weights of mice at day 10. **(c)** The DAI scores of mice at day 10. (b, c) Significance was determined using one-way ANOVA corrected for multiple comparisons with a LSD-t test; $*P < 0.05$, $**P < 0.01$. Data are mean ± SD.

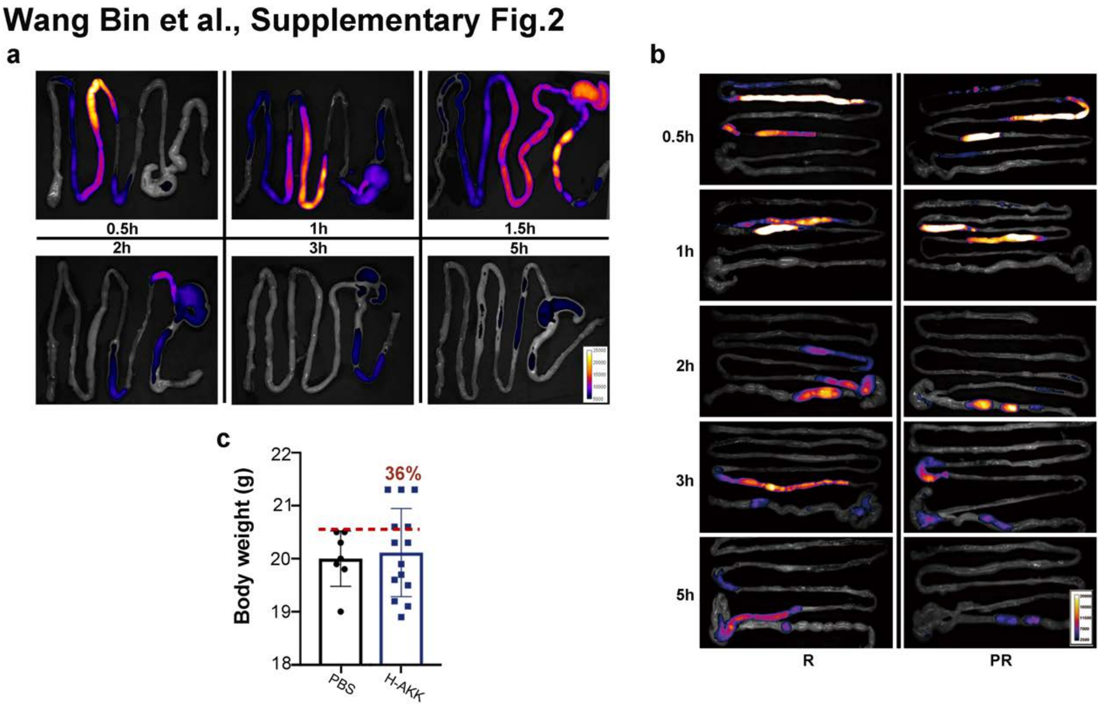

Supplementary Fig. 2 The gastrointestinal dynamics of *A. muciniphila*.

(a) The distribution of *A. muciniphila* in the gastrointestinal tract of mice with colitis in 5 h. *A. muciniphila* was labelled by DIR. **(b)** The dynamic of DIR-labelled *A. muciniphila* in R and PR group. Images were representative of three biological replicates. **(c)** The body weights of mice treated by PBS or *A. muciniphila* of high concentration at day 10.

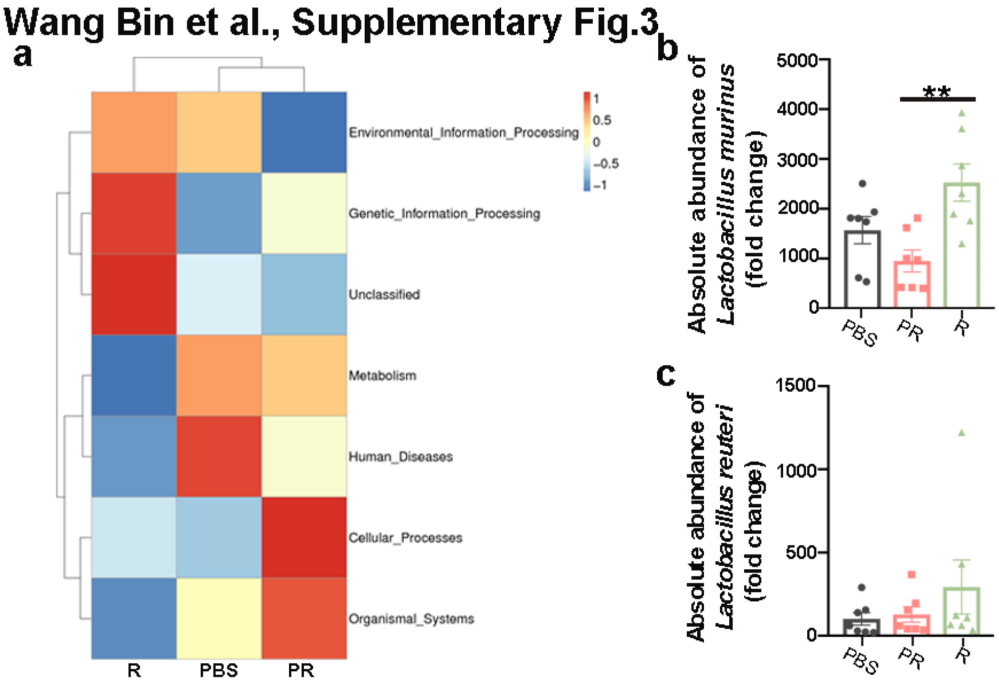

Supplementary Fig. 3 The colonization intensity of *A. muciniphila* affects the gut microecology of mice.

(a) The difference heat-map of gut bacteria functions. **(b, c)** Statistical results of absolute abundance of *Lactobacillus murinus* and *Lactobacillus reuteri* in the feces of mice (outliers removed). Significance is determined using Student's *t*-test; $**P < 0.01$. Data are mean ± SD.

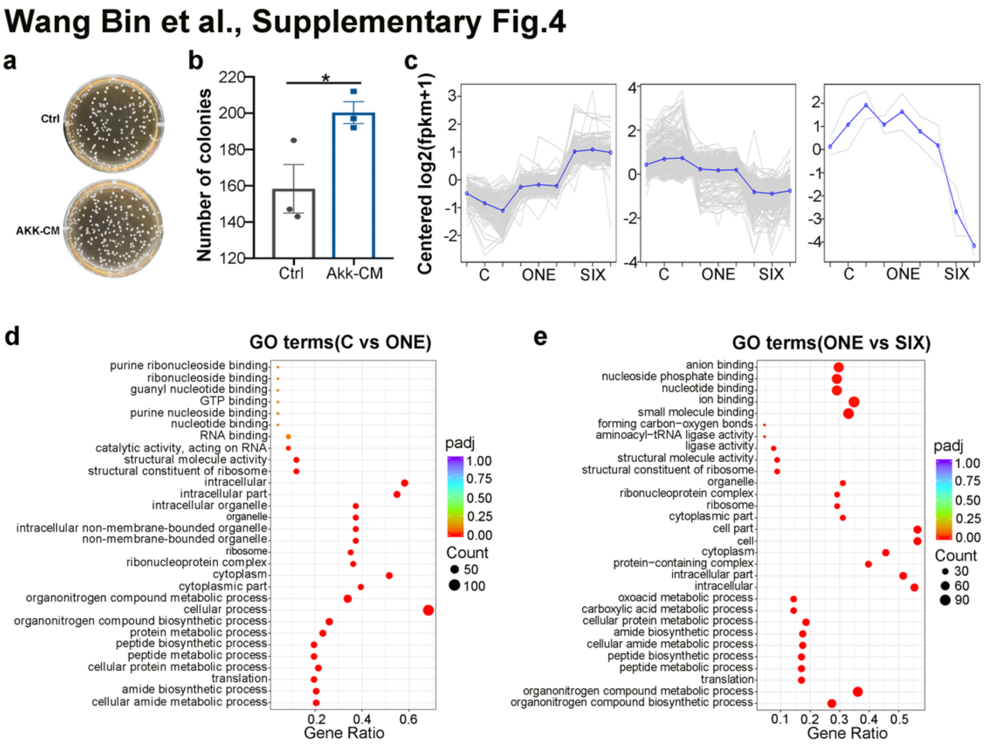

Supplementary Fig. 4 Live *A. muciniphila* reprograms gene profile of *L. murinus*.

(a) Representative pictures of colonies. **(b)** The number of colonies. Significant differences are indicated: $*P < 0.05$ by Student's *t*-test between each two cohorts. **(c)** Cluster line graphs of expression patterns of different experimental groups. Different gene sets are divided into several clusters by H-cluster, and genes in the same cluster have similar expression patterns under different processing conditions. **(d, e)** The GO function enrichment analysis of the differential genes with up-regulation in the ONE group compared with the C group (d) and in the SIX group compared with the ONE group (e). The enrichment takes padj < 0.05 as the threshold for significant enrichment.

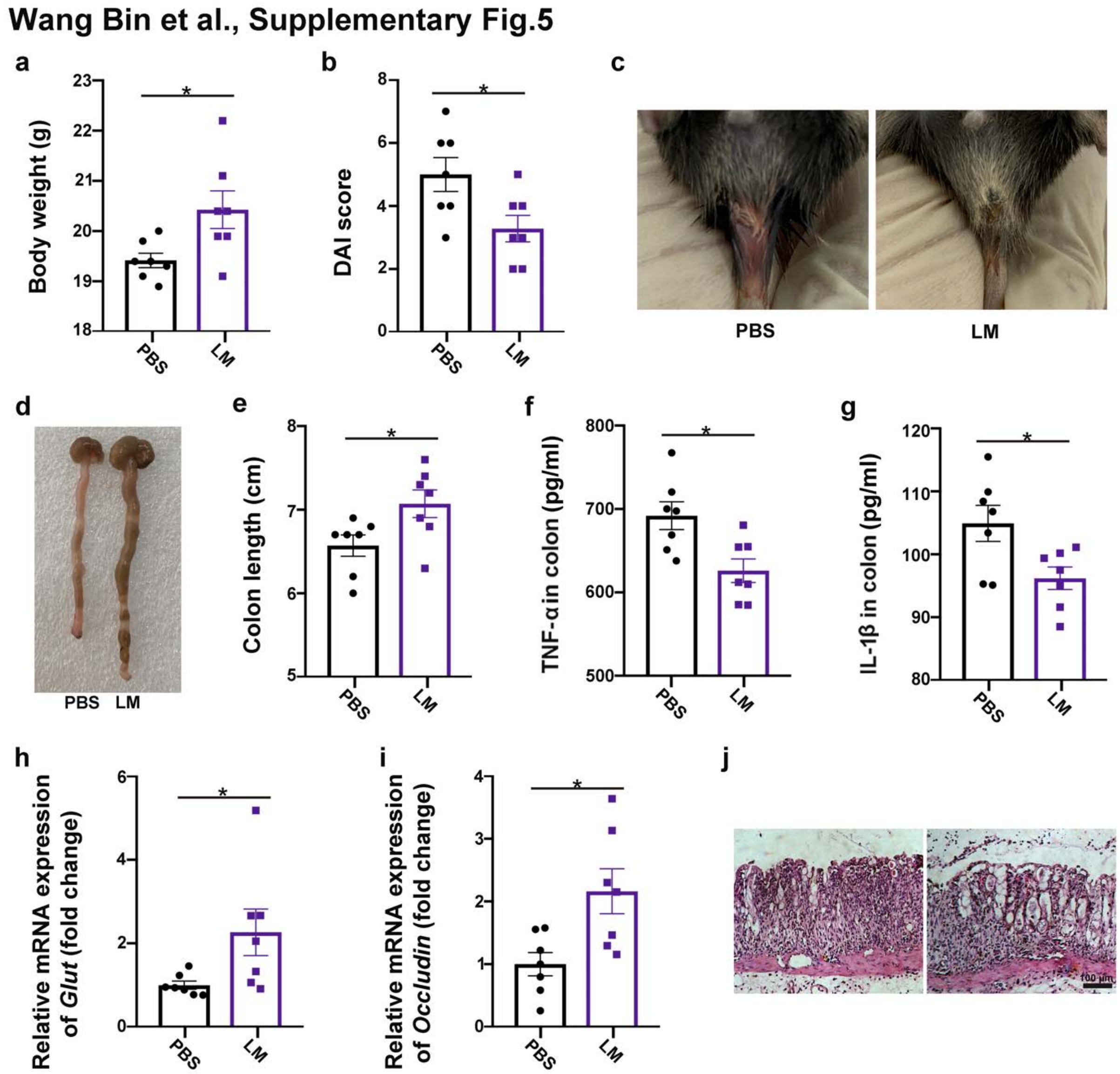

Supplementary Fig. 5 *Lactobacillus murinus* alleviates DSS-induced colitis.

Groups: PBS, mice were treated by PBS; LM, mice were treated by *L. murinus* (1×10^8 CFU/200 μl per mouse). **(a)** The body weight. **(b)** Statistical results of DAI score. **(c)** Representative photographs of rectal bleeding of IBD mice on day 10. **(d)** Representative photographs of colons of mice. **(e)** Statistical results of colon length of mice. **(f, g)** The levels of TNF-α and IL-1β in inflamed colon of mice were measured by ELISA. **(h, i)** The relative mRNA expression levels of *Glut* and *Occludin* were examined in colon tissues from mice by qRT-PCR. **(j)** Representative H&E images of colon (scar bar: 100 μm). Significance is

determined using Student’s t-test; $^*P < 0.05$.

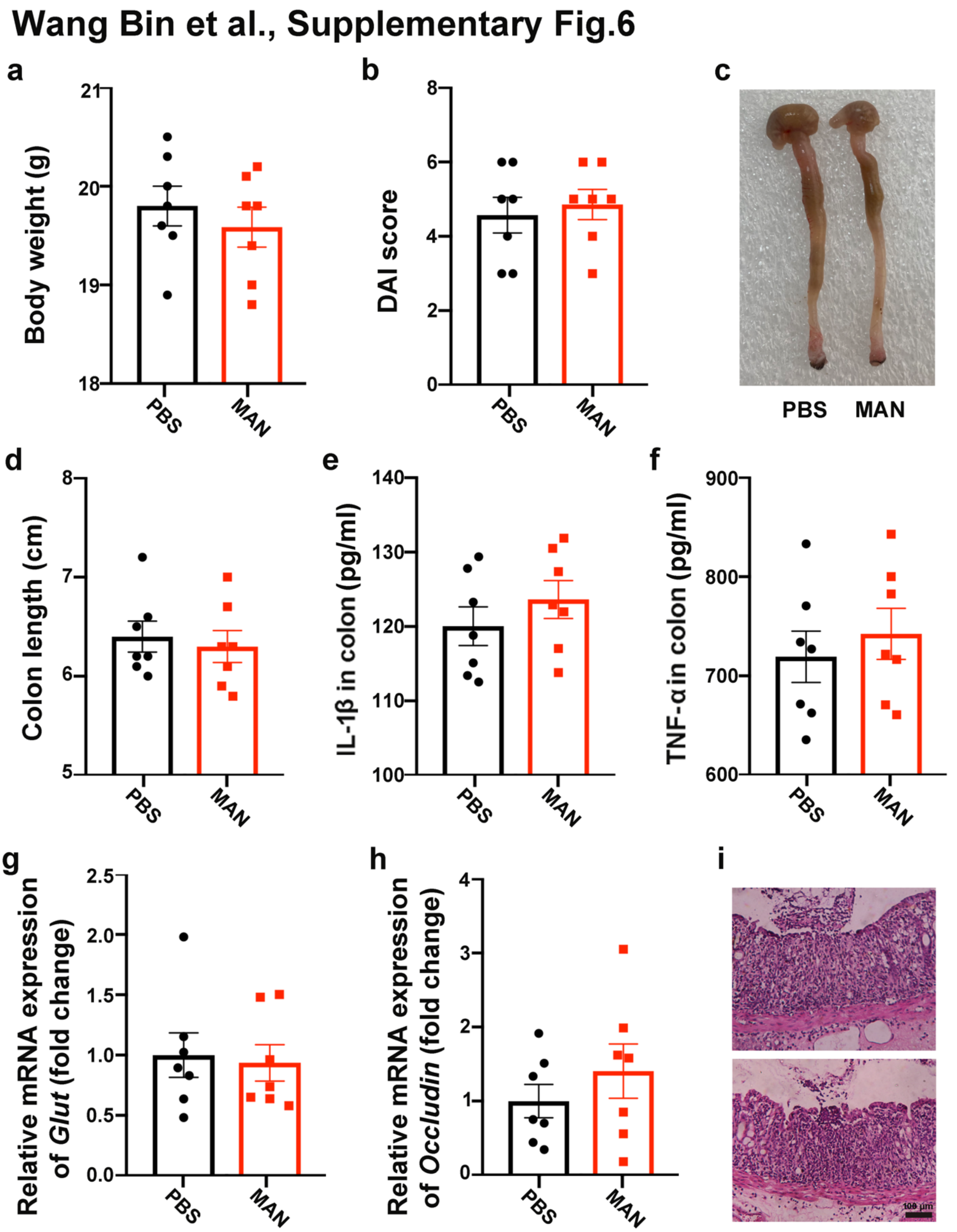

Supplementary Fig. 6 Mannose has no therapeutic impact on DSS-induced colitis.

Groups: PBS, mice were treated by PBS; MAN, mice were treated by mannose (10 μg/per

mouse). **(a)** The body weight. **(b)** Statistical results of DAI score. **(c)** Representative photographs of colons. **(d)** Statistical results of colon length. **(e, f)** The levels of IL-1β and TNF-α in inflamed colon of mice were measured by ELISA. **(g, h)** The relative mRNA expression levels of *Glut* and *Occludin* were examined in colon tissues from mice by qRT-PCR. **(i)** Representative H&E images of colon (scar bar: 100 μm). Significance is determined using Student's *t*-test.

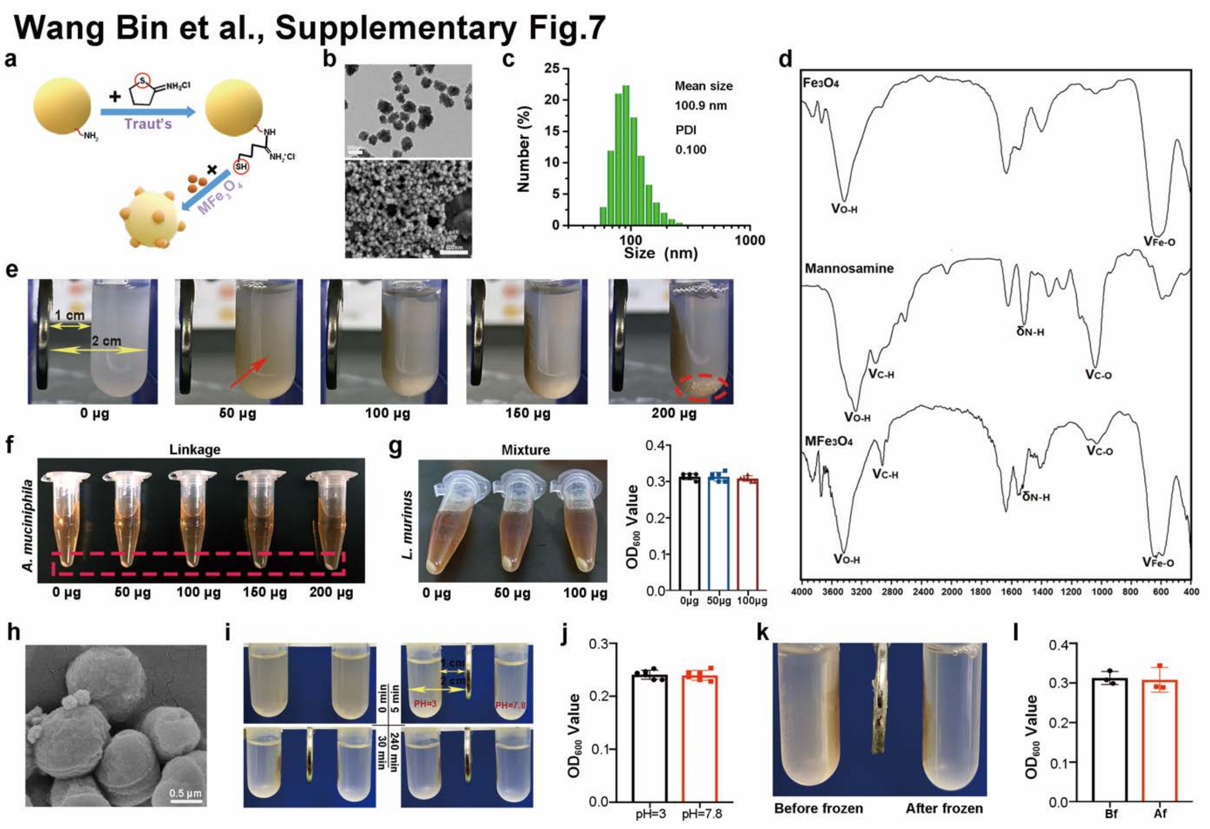

Supplementary Fig. 7 Preparation and characterization of magnetic *A. muciniphila* - AKK@MFe$_3$O$_4$.

(a) Preparation scheme of AKK@MFe$_3$O$_4$, the red circles represent the reaction site. **(b)** TEM (up) and SEM images (down) of Fe$_3$O$_4$ nanoclusters. **(c)** Size distributions of Fe$_3$O$_4$ nanoclusters. **(d)** The FT-IR spectra of Fe$_3$O$_4$, D-mannosamine and MFe$_3$O$_4$ nanoclusters. **(e)** The magnetic attraction images of AKK@MFe$_3$O$_4$ with various concentrations of MFe$_3$O$_4$

decoration under a gentle shaking. **(f)** Representative photographs of precipitate agglomerate of *A. muciniphila* that cultured for 12 h. *A. muciniphila* was linked with MFe_3O_4 of different concentration for 6 h. **(g)** Representative photographs of precipitate agglomerate and OD_{600} value of *L. murinus* that cultured for 24 h. *L. murinus* was mixed with MFe_3O_4 of different concentration for 6 h. **(h)** The SEM images of AKK@MFe_3O_4 after culturing in anaerobic environment for 6 h (scar bar: 0.5 μm). **(i)** The magnetic attraction images of AKK@MFe_3O_4 under simulated gastric juice (pH 3.0) and intestinal fluid (pH 7.8) at various times. **(j)** The OD_{600} value of AKK@MFe_3O_4 following incubating in simulated gastrointestinal environment of different pHs for 6 h. **(k, l)** The magnetic attraction images (k) and OD_{600} value (l) of AKK@MFe_3O_4 before freezing (Bf) and after thawing (Af) (stored in PBS containing 25% glycerol).

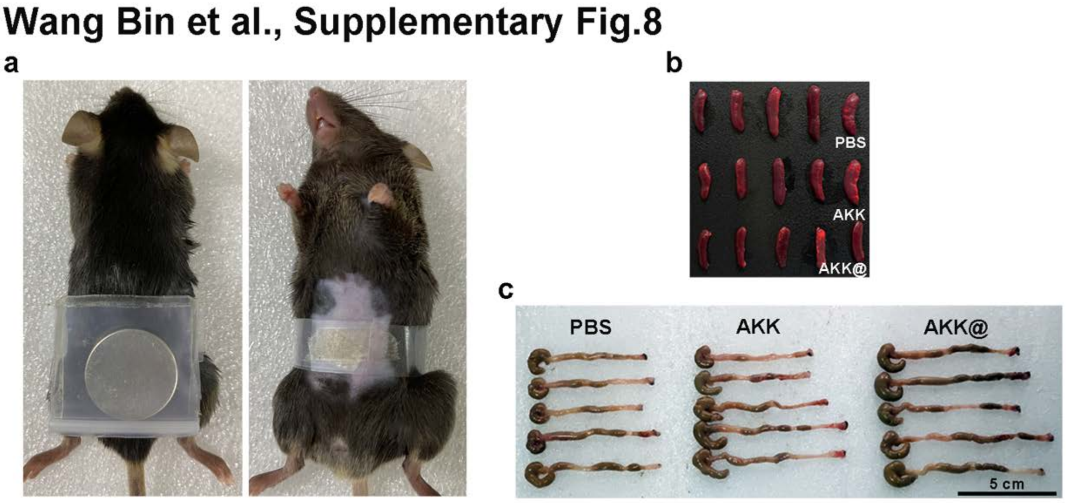

Supplementary Fig. 8 The magnetic *A. muciniphila* improves the therapeutic efficacy to colitis.

(a) Mouse with the magnetic device. **(b)** Representative photographs of spleens of mice. **(c)** Representative photographs of colons of mice (scar bar: 5 cm).

Wang Bin et al., Supplementary Fig.9

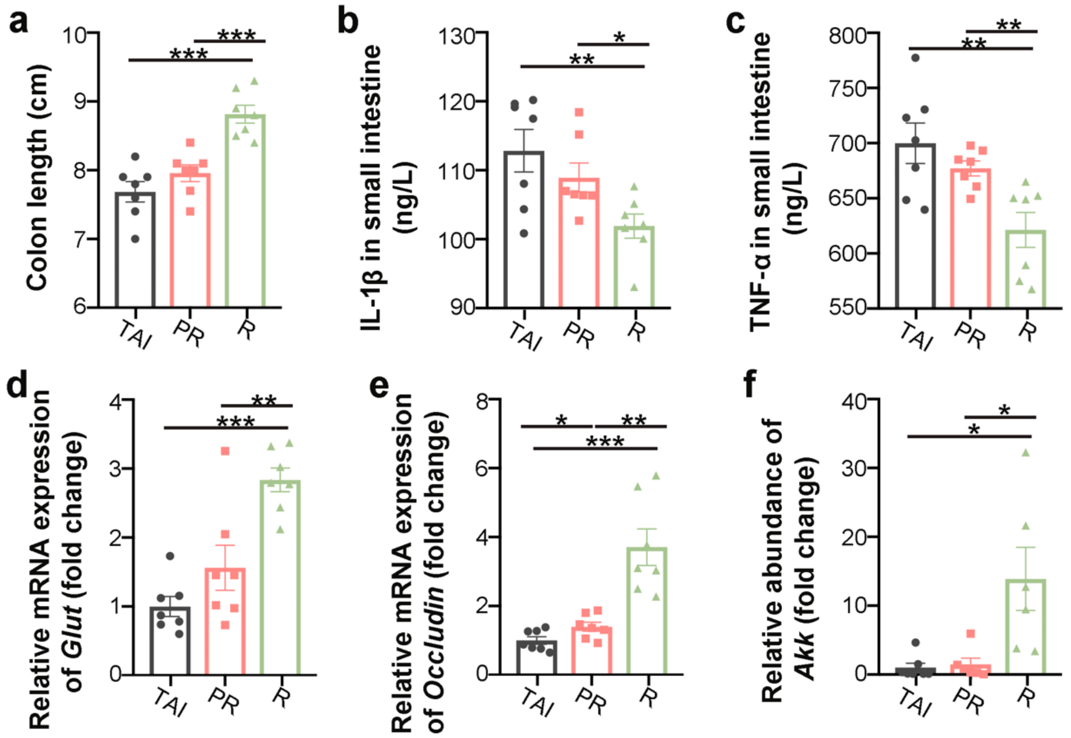

Supplementary Fig. 9 The colonization of *A. muciniphila* correlates to therapeutic efficacy against radiation-induced small intestinal injury.

All experimental mice were received 12 Gy γ-ray total abdominal irradiation (TAI). Groups: TAI, the mice were administrated with PBS; PR, poor response to *A. muciniphila*; R, response to *A. muciniphila*. **(a)** Statistical result of colon length of mice. **(b, c)** The levels of IL-1β and TNF-α in small intestines of mice were measured by ELISA. **(d, e)** The relative mRNA expression levels of *Glut* and *Occludin* were examined in small intestines from mice by qRT-PCR. **(f)** The relative abundance of *A. muciniphila* in the small intestinal mucosa was detected by q-PCR. Significance was determined using one-way ANOVA corrected for multiple comparisons with a LSD-t test; $*P < 0.05$, $**P < 0.01$, $***P < 0.001$. Data are mean ± SD.

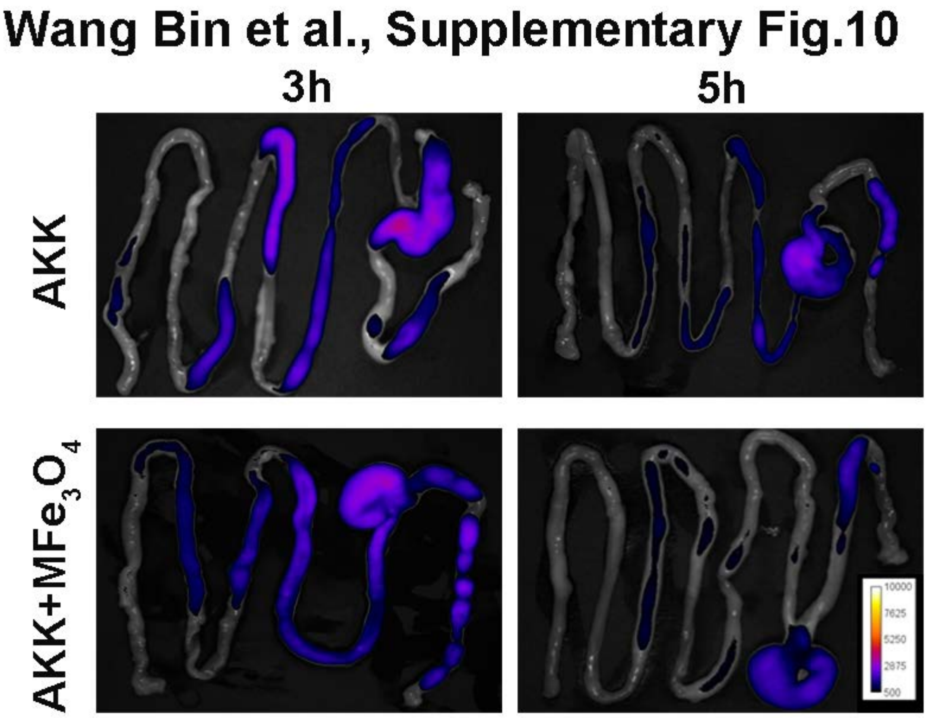

Supplementary Fig. 10 The retention of *A. muciniphila* in AKK and AKK+ groups (single gavage treatment).

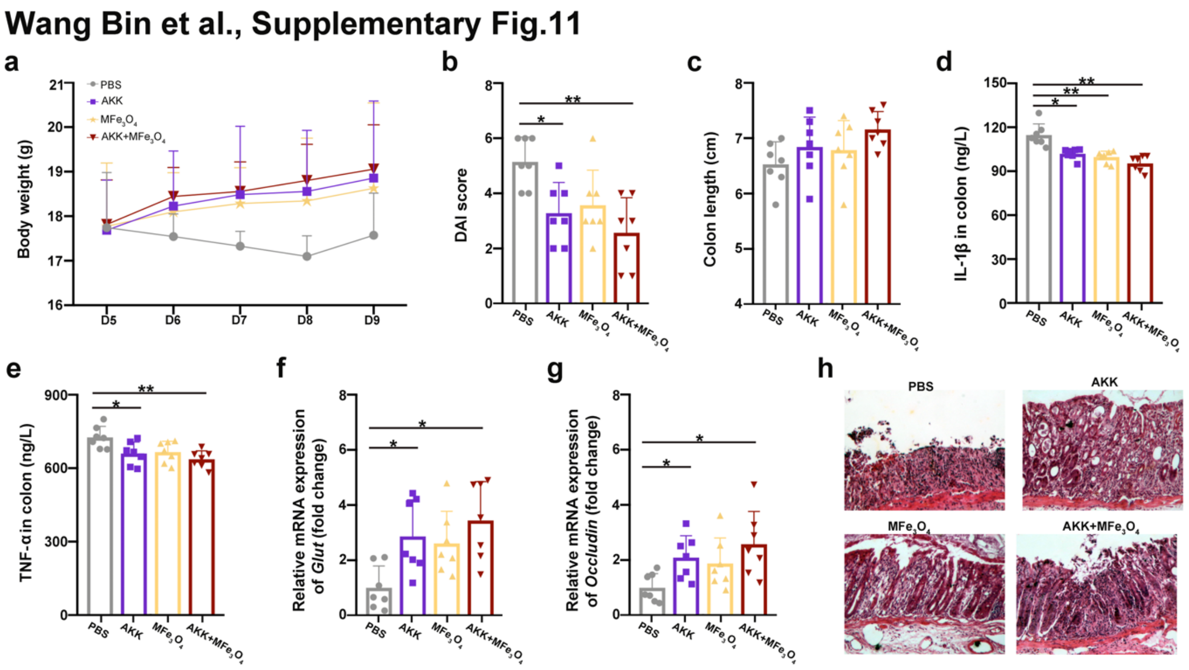

Supplementary Fig. 11 The therapeutic effects of PBS, *A. muciniphila*, MFe_3O_4 and AKK+MFe_3O_4 on DSS-induced colitis.

(a) Daily body weight of the mice. **(b)** The DAI score of colitis assessed on day 10. **(c)**

Statistical results of length of colon. **(d, e)** The levels of IL-1β and IL-6 in colons of mice were measured by ELISA. **(f, g)** The relative mRNA expression levels of *Glut* and *Occludin* were examined in colon tissues from mice by qRT-PCR. **(h)** Representative H&E images.

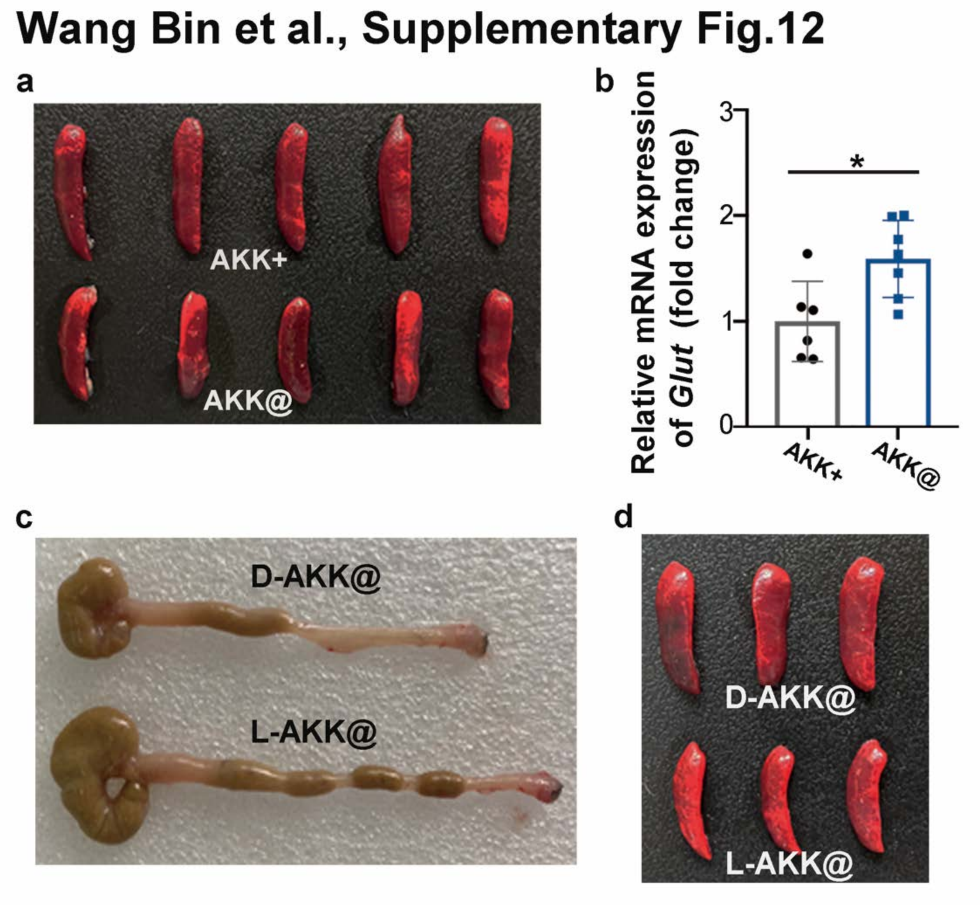

Supplementary Fig. 12 *A. muciniphila* colonization dictates therapeutic efficacy of AKK@MFe$_3$O$_4$ to DSS-induced colitis.

(a) Representative photographs of spleens of mice. **(b)** The relative mRNA expression levels of *Glut* were examined in colon tissues from mice by qRT-PCR. **(c)** Representative photographs of colons of mice (scar bar: 5 cm). **(d)** Representative photographs of spleens of mice.

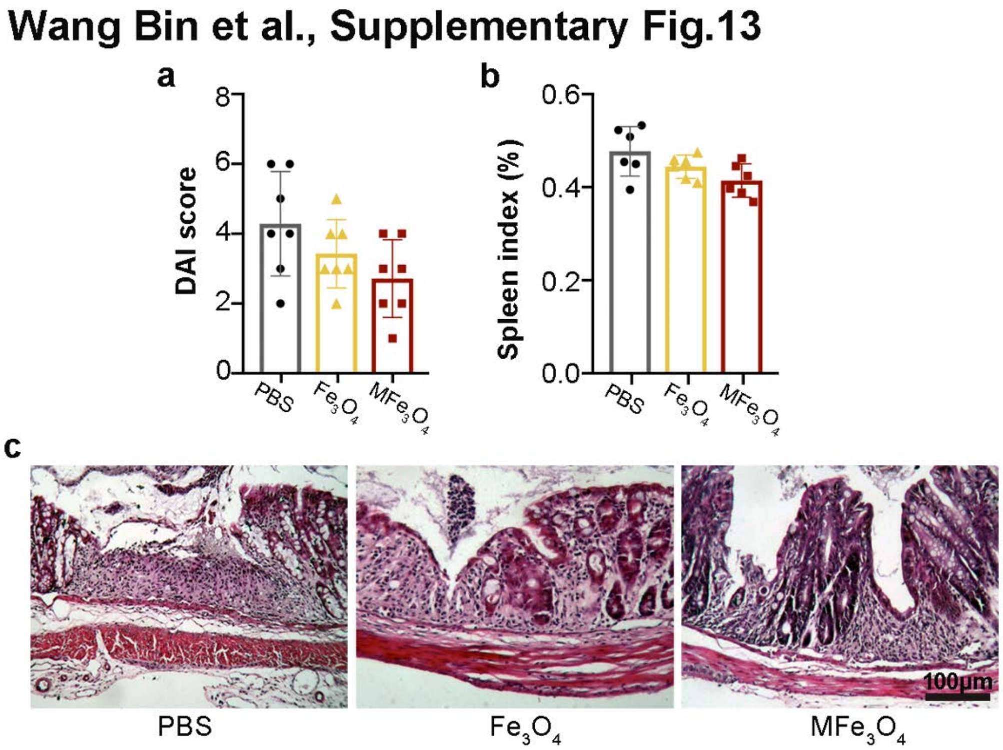

Supplementary Fig. 13 Fe_3O_4 and MFe_3O_4 improve the recovery of DSS-induced colitis.

(a) The DAI score. **(b)** Statistical results of spleen index. **(c)** Representative H&E images (scar bar: 100 μm).

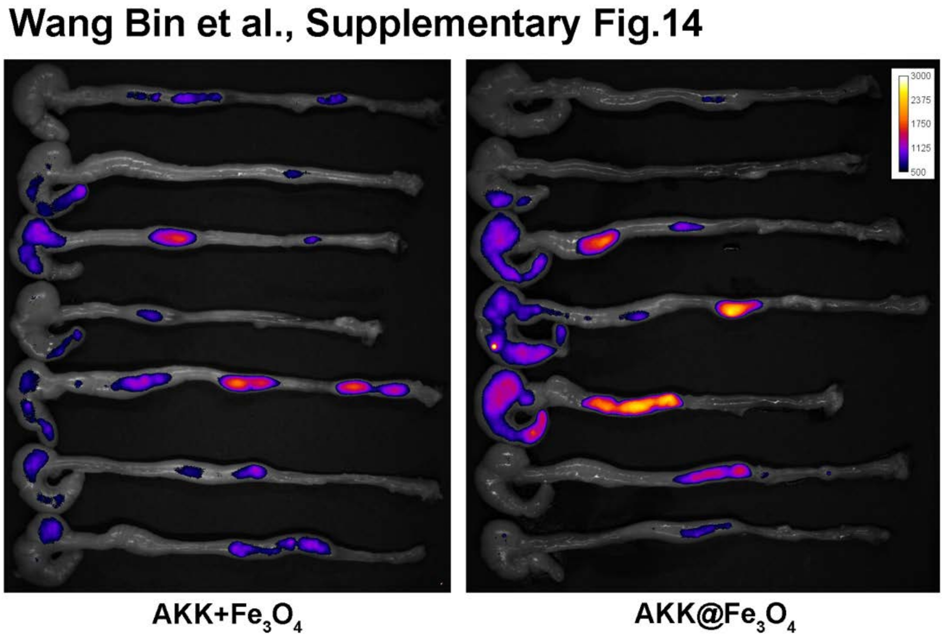

Supplementary Fig. 14 AKK@MFe_3O_4 was excreted from the intestine of mice eventually.

The mice with colitis were orally administrated with AKK+Fe_3O_4 or AKK@MFe_3O_4 for three days respectively and *A. muciniphila* was labelled by DIR. The fluorescence images of colons were obtained at 24 h following the last treatment.
